# Inosine enhances tumor mitochondrial respiration by inducing Rag GTPases and nascent protein synthesis under nutrient starvation

**DOI:** 10.1038/s41419-023-06017-2

**Published:** 2023-08-02

**Authors:** Mei-Xin Li, Xiao-Ting Wu, Wen-Qiang Jing, Wen-Kui Hou, Sheng Hu, Wei Yan

**Affiliations:** Hubei Key Laboratory of Cell Homeostasis, College of Life Sciences, TaiKang Center for Life and Medical Sciences, Wuhan University, Wuhan, Hubei 430072 China

**Keywords:** Cell biology, Cancer

## Abstract

Metabolic heterogeneity of tumor microenvironment (TME) is a hallmark of cancer and a big barrier to cancer treatment. Cancer cells display diverse capacities to utilize alternative carbon sources, including nucleotides, under poor nutrient circumstances. However, whether and how purine, especially inosine, regulates mitochondrial metabolism to buffer nutrient starvation has not been well-defined yet. Here, we identify the induction of 5′-nucleotidase, cytosolic II (NT5C2) gene expression promotes inosine accumulation and maintains cancer cell survival in the nutrient-poor region. Inosine elevation further induces Rag GTPases abundance and mTORC1 signaling pathway by enhancing transcription factor SP1 level in the starved tumor. Besides, inosine supplementary stimulates the synthesis of nascent TCA cycle enzymes, including citrate synthesis (CS) and aconitase 1 (ACO1), to further enhance oxidative phosphorylation of breast cancer cells under glucose starvation, leading to the accumulation of iso-citric acid. Inhibition of the CS activity or knockdown of ACO1 blocks the rescue effect of inosine on cancer survival under starvation. Collectively, our finding highlights the vital signal role of inosine linking mitochondrial respiration and buffering starvation, beyond serving as direct energy carriers or building blocks for genetic code in TME, shedding light on future cancer treatment by targeting inosine metabolism.

## Introduction

Cancer metabolism has been studied extensively over the past two decades, and it is widely accepted that oncogenic transformation could cause cancer cells to adopt a well-characterized metabolic phenotype that can profoundly influence the tumor microenvironment (TME) [1–4]. The metabolic heterogeneity of TME results from the unequal supply of oxygen and nutrients by the tumor vasculature across the tumor bulk [5, 6]. This poor vascular exchange results in nutrient limitation in the TME, while the bioenergetic demands of rapidly proliferating cancer compete for nutrients [7, 8]. Strategies for cell metabolism alteration would offer promising opportunities for cancer therapies, and identifying targets that suppress or alter cancer metabolism to improve the TME nutrient availability would benefit maximizing the efficacy of cancer therapies [2, 9, 10]. Metabolic reprogramming is one of the hallmarks of cancer to serve as an adaptive mechanism by which fast-growing cancer cells adapt to their increasing energy demands [7, 11]. To fulfill these needs, cancer cells undergo metabolic rewiring to meet their bioenergetic, biosynthetic, and redox demands relying on glucose and glutamine as major carbon and nitrogen source but also extending to nucleotides [12].

Nucleotides and their derivatives act as energy carriers to drive enzymatic reactions to exert active biochemical and cellular signaling functions in tumor biology beyond the building blocks of the genetic code [13, 14]. Resided in the central nature of nucleotides in cellular function, elevated purine abundance depending both salvage from existing bases and de novo biosynthesis [13, 15]. Under normal physiological conditions, most of the purine pool is generated by the salvage pathway with the nucleic acid breakdown process, inducing the release of free purine nucleobases in the form of adenine, guanine, and the hypoxanthine base of inosine 5′-monophosphate (IMP) [16]. The inadequacy of intracellular purine nucleotide pools impacts cell physiological action. Reducing intracellular purines re-routes the metabolic flux from glycolysis into de novo serine synthesis to trigger an epithelial–mesenchymal transition (EMT) and cancer cell migration [17]. Incremental evidence demonstrates that the elevation of purine de novo biosynthesis through particular regulation of several metabolism pathways, such as the mammalian target of rapamycin complex 1 (mTORC1) signaling pathway, supports the proliferation and invasion of cells via metabolic reprogramming [18].

The mTORC1 integrates signals from growth factors and nutrients through the Rheb and Rag GTPases residing on the lysosomal surface to control biosynthetic processes, including protein, lipid, and nucleic acid synthesis [19–22]. Unlike how Rheb activates on mTORC1 by sensing growth factors, the Rag GTPases regulate mTORC1 translocation to the lysosomal surface in response to the levels of nutrients [23]. The Rag GTPases are heterodimers formed by an RRAGA or RRAGB subunit binding to either RRAGC or RRAGD subunit [24, 25]. Depletion of cellular purines inhibits mTORC1, and restoration of intracellular adenine nucleotides via the addition of exogenous purine nucleobases or nucleosides acutely reactivates mTORC1 [26]. Simultaneously, mTORC1 phosphorylates some essential proteins, thereby promoting mitochondrial DNA replication, fusion, and oxidative phosphorylation but inhibiting glycolysis or autophagy [27]. Here, we show inosine is elevated in the tumor core (TC) region and induces a cluster of essential TCA cycle enzymes by stimulation of mTORC1 signaling pathway mediated catabolic activity to meet the increased demand of biomolecules and energy for buffering cancer cell survival under glucose starvation. Therefore, a better understanding of the mechanistic links between cancer metabolism may ultimately lead to precision treatments for human cancer, and targeting inosine metabolism might be a promising strategy for clinical application.

## Results

### Elevated inosine promotes BC survival under glucose starvation in the TC

**Fig. 1:**
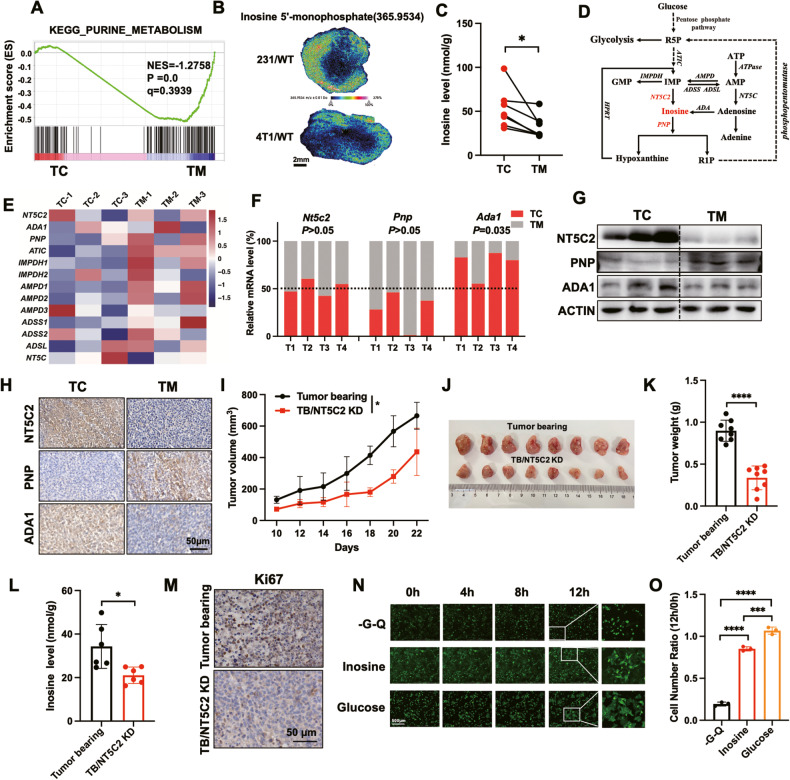
Elevation of inosine level promotes BC survival under glucose starvation. **A** MDA-MB-231 tumor tissues from the core and margin regions were separated to subject to RNA-seq and GSEA analysis, showing enrichment of genes related to the indicated pathway (*n* = 3 mice per group). **B** Representative mass spectrometry imaging for Inosine-5′-monophosphate abundance in MDA-MB-231 and 4T1 wild-type tumors from NSG mice and Balb/C mice. Scale bar, 2 mm. **C** Tissues from core and margin regions of 4T1 xenografted tumors were used for inosine detection. Inosine concentrations were measured by ELISA kit (paired two-tailed Student’s *t*-test, *n* = 7 mice per group). **D** Schematic diagram depicting inosine metabolic pathway. **E** The level of genes in purine pathway gene sets in the core and margin regions of MDA-MB-231 xenografted tumors are shown as a heat map. **F** Relative *Nt5c2*, *Pnp*, and *Ada1* RNA levels in core and margin regions of 4T1 cells xenografted tumors determined by RT-qPCR (paired two-tailed Student’s *t*-test, *n* = 4 mice per group). **G** 4T1 tumor tissues were harvested from Balb/C mice. Tissues from the core and margin regions were separated to collect whole cell lysates, followed by western blots analysis. Indicated protein levels were assessed by western blots. **H** Representative IHC images showing indicated protein staining of core and margin regions tissues separated from 4T1 tumors. Scale bar, 50 μm. **I** Tumor volume followed in indicated xenografted mice (two-way ANOVA, *n* = 4 mice per group). **J** Representative tumor images of indicated xenografted tumors in Balb/C mice were shown (*n* = 8 mice per group). **K** Tumor weight was monitored at the end of the experiment in indicated xenografted mice (unpaired two-tailed Student’s *t*-test, *n* = 8 mice per group). **L** Tissues of 4T1 and 4T1/NT5C2 KD cells xenografted tumors were used for inosine detection. Inosine concentrations were measured by ELISA kit (paired two-tailed Student’s *t*-test, *n* = 6 mice per group). **M** Representative IHC images showing Ki67 staining of core regions tissues separated from 4T1 and 4T1/NT5C2 KD tumors. Scale bar, 50 μm. **N** MDA-MB-231 labeled Lck-GFP (231/Lck-GFP) cells were treated with glucose–glutamine-deficient medium (-G-Q) or supplemented with inosine or glucose. The fluorescent images were taken at the indicated time. Scale bar, 500 μm. **O** Quantification of the fold change of the cell numbers for (**N**) (one-way ANOVA, *n* = 3 biological replicates).

To better understand the genetic regulation of metabolic heterogeneity, we performed RNA-seq for both TC and tumor margin (TM) zone from mouse primary breast cancer (BC) tissues. We found significantly downregulated purine metabolism in TC (Fig. 1A). Consistently, we examined the purine levels for tumors from mice xenografted with human (231/WT) and murine (4T1/WT) triple-negative BC cells by employing mass spectrometry imaging (MSI). The abundances of inosine 5′-monophosphate (IMP), adenosine 5′-monophosphate, and hypoxanthine were significantly decreased in TC than TM in both 231/WT and 4T1/WT tumors, but with mere alteration of xanthosine 5′-phosphate, xanthosine, ATP and adenosine levels (Fig. 1B and S1A). However, there was significantly elevated inosine accumulation in the TC region of 4T1 tumors (Fig. 1C). Similarly, the accumulation of inosine was observed in the TC of the colon (SW480), liver (Hep3B) and lung (A549) primary tumors (Fig. S1C). Besides, we also detected higher ATP levels in 4T1 tumor core regions and MDA-MB-231 cells with inosine treatment under nutrient starvation compared to their control groups separately (Figs. S1D, E). According to the metabolic pathway for IMP conversion, the cytosolic 5′ nucleotidase II (NT5C2) catalyzed the transformation of IMP to inosine (Fig. 1D). Intriguingly, RNA-Seq data demonstrated an increase of *Nt5c2* expression but purine nucleoside phosphorylase (*Pnp*) inhibition was detected in TC regions when compared to TM areas (Fig. 1E). However, there were no significant alterations of the RNA levels of *Impdh1*, *Impdh2, Hprt*, *Adsl*, *Adss*, *Atic*, *Ampd1*, and *Ampd2*, which resided in the purine pathway (Fig. S1B). Likewise, both RT-qPCR quantified RNA and protein abundance of NT5C2 was significantly increased, but with significant suppression of PNP level in TC than TM area of breast tumors (Fig. 1F, G). IHC staining also demonstrated more NT5C2 and ADA1 but lower PNP expressed in the TC regions (Fig. 1H). To investigate the biological function of inosine in the TC region, we constructed a 4T1/NT5C2 knockdown cell line (4T1/NT5C2 KD) and found the intracellular inosine level in 4T1/NT5C2 KD cells was significantly reduced when compared with 4T1 WT cells (Fig. S1F, G). Moreover, compared with 4T1 tumor-bearing mice, 4T1/NT5C2 KD tumor-bearing mice displayed retarded tumor growth and smaller tumor volume but steady weight gain (Fig. 1I–K and S1H). Similarly, the concentration of inosine in TC of 4T1/NT5C2 KD tumor was significantly decreased compared with the control group (Fig. 1L). Ki67 staining assay evidenced less proliferation in TC of 4T1/NT5C2 KD tumor (Fig. 1M). In addition, we injected inosine into the TC region, and a larger tumor size and more tumor weight were detected in inosine-injected tumors compared to the control group (Figs. S1I, J). Ki67 staining assay also confirmed more proliferation in inosine-injected tumors compared to the control group (Fig. S1K). Besides, we also observed MDA-MB-231 cells cultured under both glucose and glutamine-deficient medium displayed almost fivefold downregulation of the survival rate when compared to the cells cultured with additional inosine or glucose supplementary (Fig. 1N, O). Overall, those results suggested that elevation of inosine could buffer nutrient starvation to promote cancer cell survival.

### Inosine enhances the mTORC1 signaling pathway by inducing Rag GTPases expression

**Fig. 2:**
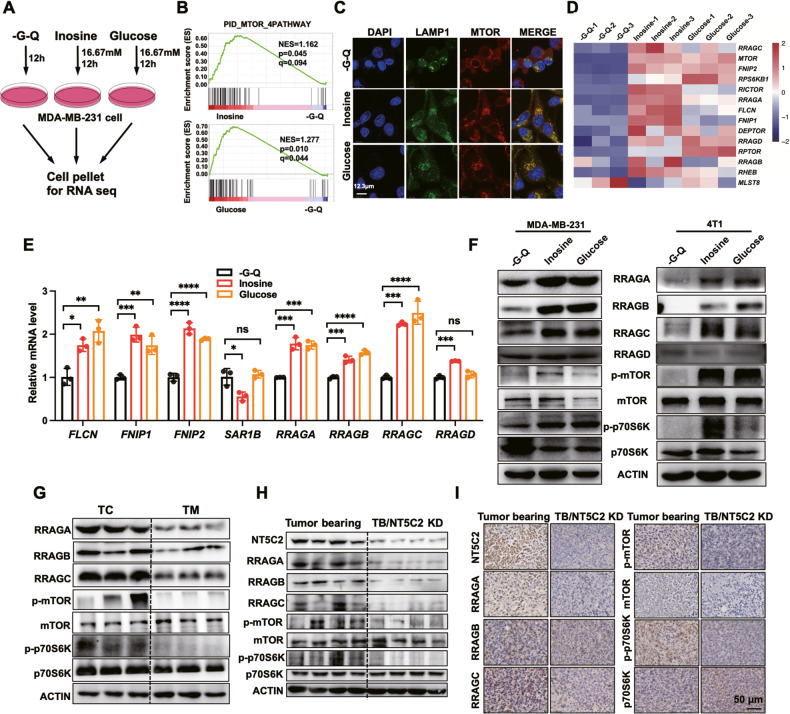
Inosine enhances the mTORC1 signaling pathway by inducing Rag GTPases. **A** Schematic diagram of MDA-MB-231 cells treated with glucose–glutamine-deficient medium or supplemented with inosine or glucose for 12 h.The cell pellets were collected and subjected to RNA-seq. **B** MDA-MB-231 cells were subjected to RNA-seq and GSEA as described in the figure above, showing enrichment of genes related to indicated pathways (*n* = 3 biological replicates). **C** Colocalization of mTOR (red) and LAMP1 (green) in MDA-MB-231 cells (glucose–glutamine-deficient medium (-G-Q) or supplemented with inosine or glucose). Scale bar, 12.3 μm. **D** The level of genes in mTOR pathway gene sets in MDA-MB-231 cells (-G-Q or supplemented with inosine or glucose) are shown as a heat map. **E** Relative RNA levels were detected by RT-qPCR in MDA-MB-231 cells treated with glucose–glutamine-deficient medium (-G-Q) or supplemented with inosine or glucose (one-way ANOVA, *n* = 3 biological replicates). **F** Western blots showing the protein expression levels in MDA-MB-231 and 4T1 cells treated with glucose-glutamine-deficient medium (-G-Q) or supplemented with inosine or glucose. **G** Western blots showing the protein expression levels in the core and margin regions, tissues were separated from 4T1 tumors. **H** Western blots showing the protein expression levels in the core regions, tissues were separated from 4T1 tumors and 4T1/NT5C2 KD tumors. **I** Representative IHC images showing indicated protein staining of core regions, tissues were separated from 4T1 tumors and 4T1/NT5C2 KD tumors. Scale bar, 50 μm.

To further investigate the underlying mechanism of how inosine promoted tumor survival, we treated MDA-MB-231 cells under glucose and glutamine starvation, with additional inosine and glucose supplementary, respectively (Fig. 2A). GSEA and KEGG enrichments demonstrated significant upregulation of mTORC1 signaling pathway upon inosine or glucose supplementary when compared to glucose and glutamine starvation (Fig. 2B and S2A). Besides, the ribosome signaling pathway was significantly enriched upon inosine treatment, indicating that mRNA translation was induced by inosine (Fig. S2B). Intriguingly, inosine also stimulated mTORC1 localization to lysosomes compared with MDA-MB-231 cells under starvation conditions (Fig. 2C). All these results supported that inosine promoted the activation of the mTORC1 pathway, which was consistent with a previous report [26]. Different from Rheb-mediated mTORC1 regulation, we found inosine enhanced the expression of Rag GTPases, including *RRAGA*, *RRAGB*, *RRAGC*, and *RRAGD* (Fig. 2D). Simultaneously, upregulation of *FLCN*, *FNIP1*, and *FNIP2* but decreased *SAR1B* expression residing in the mTORC1 signaling pathway were also detected (Fig. 2E and S2C). There was a remarkable elevation of RRAGA, RRAGB, RRAGC, phosphorylated mTOR, and S6K but undetectable alteration of the RRAGD protein level in MDA-MB-231 and 4T1 cells (Fig. 2F). Additionally, the proteins residing in the Rag GTPases-mediated mTORC1 pathway were also elevated in TC, where there was more inosine accumulation (Fig. 2G). Similar patterns were observed in SW480 and Hep3B xenografted tumors core region compared with their relative tumors margin region (Fig. S2D). However, this phenomenon was totally reversed in 4T1/NT5C2 KD cells xenografted tumors (Figs. 2H, I). We injected inosine into the TC area and observed that proteins of Rag GTPases mediated mTORC1 pathway significantly upregulated in the mice that received inosine injection compared to the control group in the 4T1 cells xenografted tumors in Balb/C mice (Fig. S2E, F). To further verify whether inosine directly acted on mTORC1 or mediated through downstream catabolic derivatives, we constructed MDA-MB-231 with PNP knockdown cell line (Fig. S2G). Obviously, the mTORC1 pathway can be activated and promote most cell survival either by PNP knockdown or by Forodesine, a PNP inhibitor (Fig. S2H–K). To investigate whether cell surface adenosine receptors got involved in the inosine/mTORC1 axis, we constructed MDA-MB-231 with ADORA1 and ADORA2A knockdown cell line and found that inosine still activated the mTORC1 pathway and promoted cell survival even when ADORA1 and ADORA2A were knocked down or treated with their inhibitor SCH58261 [28] (Fig. S2L–P). Collectively, inosine activated the mTORC1 signaling pathway via upregulating Rag GTPases expression. This effect was triggered by inosine rather than its downstream derivative metabolites or any crosstalk with cell surface receptors.

### Inosine activates the Rag GTPases-mediated mTORC1 pathway by enhancing SP1 expression

**Fig. 3:**
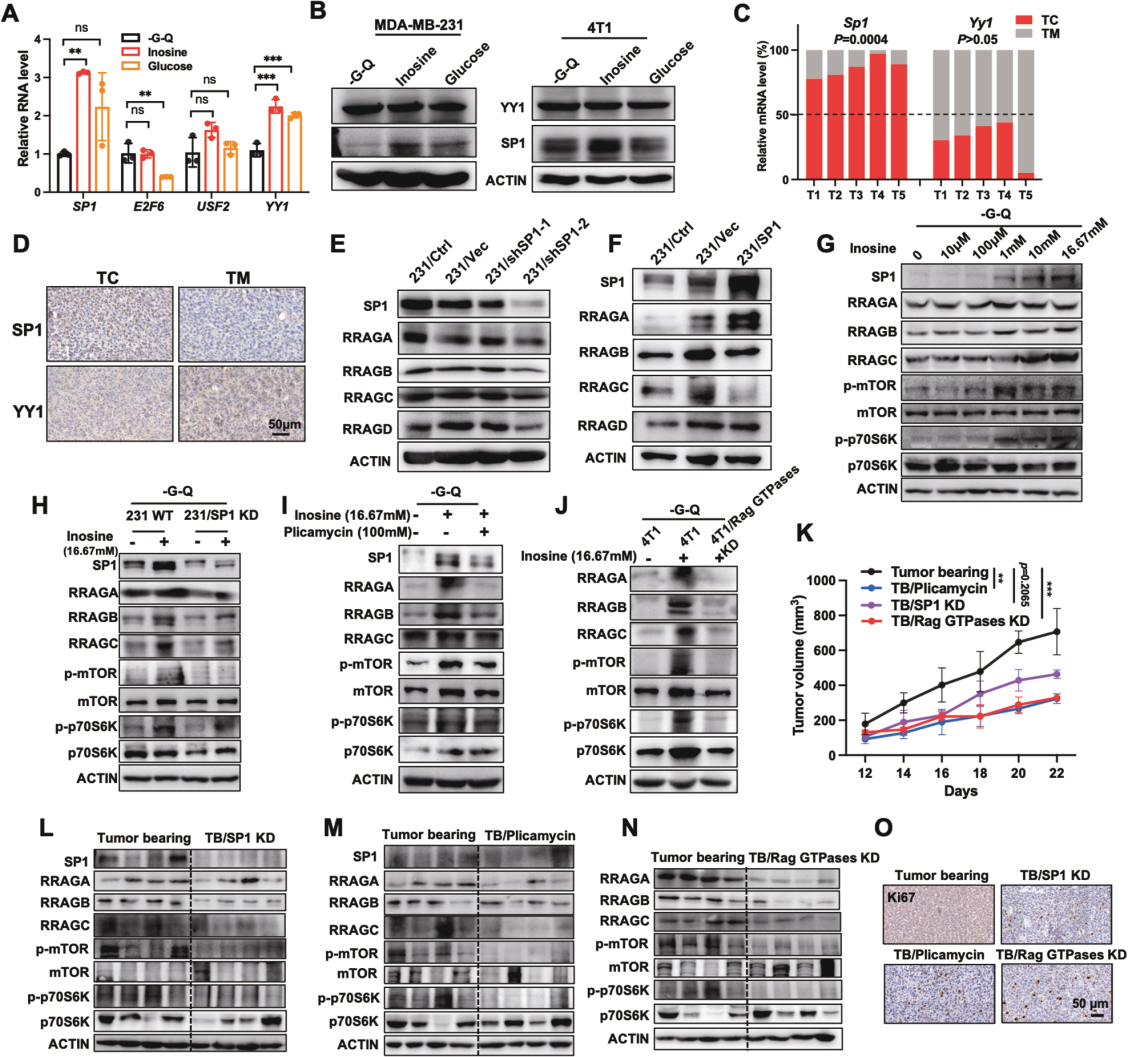
Inosine induces Rag GTPases expression by elevating the level of SP1. **A** Relative RNA levels were detected by RT-qPCR in MDA-MB-231 cells treated with glucose–glutamine-deficient medium (-G-Q) or supplemented with inosine or glucose (one-way ANOVA, *n* = 3 biological replicates). **B** Western blots showing the protein expression levels in MDA-MB-231 and 4T1 cells treated with glucose–glutamine-deficient medium (-G-Q) or supplemented with inosine or glucose. **C** Quantification analysis of RNA levels in the core and margin regions tissues separated from 4T1 tumors by RT-qPCR (paired two-tailed Student’s *t*-test, *n* = 5 mice per group). **D** Representative IHC images showing indicated protein staining of core and margin regions tissues separated from 4T1 tumors. Scale bar, 50 μm. **E** Western blots showing the protein levels in MDA-MB-231/SP1 KD cells and relative control groups. **F** Western blots showing the protein expression levels in SP1 overexpressed MDA-MB-231 cells and relative control groups. **G** Western blots showing the protein levels in MDA-MB-231 cells with a dose-dependent inosine treatment. **H** Western blots showing the protein levels in MDA-MB-231/SP1 KD cells and relative control group cells treated with glucose–glutamine-deficient medium (-G-Q) or supplemented with inosine. **I** Western blots showing the protein levels in MDA-MB-231 cells treated with glucose–glutamine-deficient medium (-G-Q) or supplemented with inosine and Plicamycin for 10 h. **J** Western blots showing the protein levels in MDA-MB-231/Rag GTPases KD cells and relative control groups cells treated with glucose–glutamine-deficient medium (-G-Q) or supplemented with inosine. **K** Tumor volume followed in indicated xenografted mice (two-way ANOVA, *n* = 4 mice per group). **L** 4T1 tumors and 4T1/SP1 KD cell xenografted tumor tissues were harvested from Balb/C mice. Tissues from the core regions were separated to collect whole cell lysates, followed by western blots analysis. Indicated protein levels were assessed by western blots. **M** 4T1 tumors injected with vehicle and Plicamycin tissues were harvested from Balb/C mice. Tissues from the core regions were separated to collect whole cell lysates, followed by western blots analysis. Indicated protein levels were assessed by western blots. **N** 4T1 tumors and 4T1/Rag GTPases KD cells xenografted tumor tissues were harvested from Balb/C mice. Tissues from the core regions were separated to collect whole cell lysates, followed by western blots analysis. Indicated protein levels were assessed by western blots. **O** Representative IHC images showing Ki67 staining of core region tissues separated from indicated tumors. Scale bar, 50 μm.

To further investigate the mechanism of how inosine stimulated the expression of Rag GTPases, we searched for the potential transcription factors (TFs) by employing the two bioinformatic tools (Jaspar and TFDB) and identified *SP1* with the highest score and multiple binding sites in the promoter regions of *RRAGA*, *RRAGB*, *RRAGC*, and *RRAGD* (Supplementary Table 5). Both *YY1*, another previously reported TF involved in mTORC1 regulation [29], and *SP1* RNA levels were significantly induced in MDA-MB-231 cells upon inosine treatment (Fig. 3A). SP1 protein expression rather than other predicted transcription factors was significantly induced upon inosine supplementary (Fig. 3B). Besides, there were more *Sp1* gene expression in the TC than in the TM region of 4T1 cells xenografted tumors (Fig. 3C). Likewise, the protein level of SP1 was increased in TC than TM of 4T1 tumors (Fig. 3D). The knockdown of SP1 remarkably inhibited all the Rag GTPases, while overexpression of SP1highly upregulated RRAGA rather than RRAGB, RRAGC or RRAGD (Fig. 3E, F). To further elucidate how mTORC1 senses inosine, we treated MDA-MB-231 cells with different concentrations of inosine. The results showed that both SP1 and Rag GTPases expression increased in an inosine dose-dependent manner, as well as the activation of a mTORC1 signaling pathway (Fig. 3G). However, the knockdown of SP1 blocked the inosine-induced Rag GTPases expression and activation of the mTORC1 pathway, and inosine-induced cell proliferation was partially inhibited (Fig. 3H and S3A). Similarly, Plicamycin, an SP1 inhibitor, inhibited the activity of SP1 and blocked inosine-induced Rag GTPases-mediated mTORC1 pathway activation and cell proliferation (Fig. 3I and S3C, D). The knockdown of Rag GTPases also inhibited the activation of the mTORC1 pathway and cell proliferation triggered by inosine (Fig. 3J and S3E, F). Moreover, the growth rate of the 4T1/SP1 KD cells xenografted tumors was slower than that of the control tumor-bearing mice in the later stages (Fig. 3K, S3B and S3G, H). Strikingly, both tumor-bearing mice with SP1 inhibitor injection and 4T1/ Rag GTPases KD cell xenografted tumors were endowed with slower tumor growth rates and much smaller sizes compared with control tumor-bearing mice (Fig. 3K and S3G, H). However, there was no significant alteration in body weight between these mice (Fig. S3I). Remarkably, the knockdown of SP1 inhibited the Rag GTPases and mTORC1 pathway in TC more than that in control tumor-bearing mice (Fig. 3L and S3J). Consistently, injection of Plicamycin inhibited SP1 activity and showed the same results (Fig. 3M and S3J). Knockdown of Rag GTPases more directly blocked the activation of the mTORC1 pathway in TC compared to control tumor-bearing mice (Fig. 3N and S3J). Therefore, blocking of SP1 or Rag GTPases failed to activate the mTORC1 pathway and inhibited tumor proliferation (Fig. 3O). Collectively, inosine activated the mTORC1 signaling pathway via upregulation of SP1/Rag GTPases axis.

### Inosine enhances mitochondrial respiration by inducing the synthesis of nascent TCA cycle enzymes

**Fig. 4:**
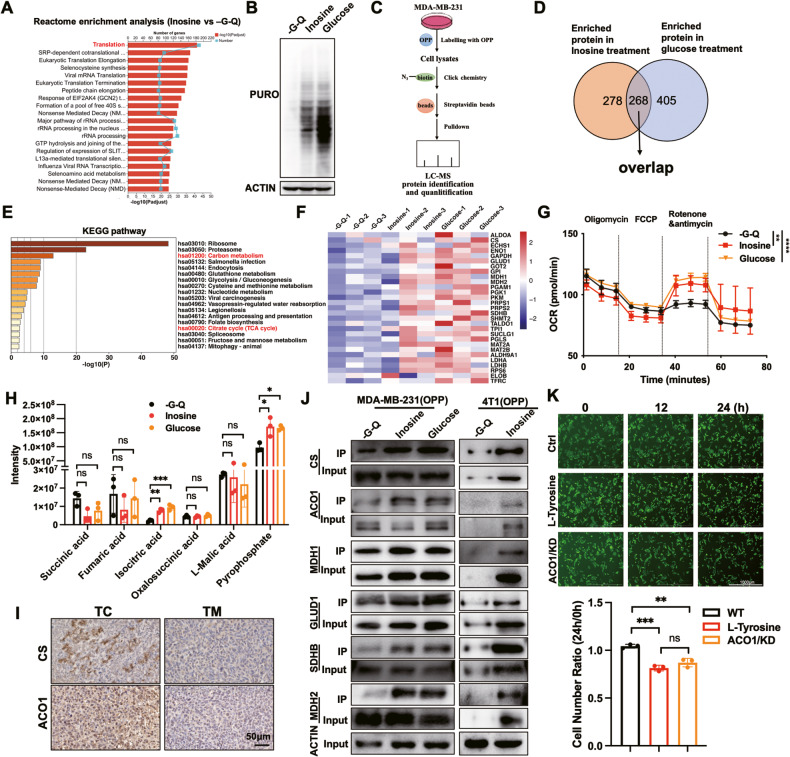
Inosine induces nascent protein synthesis to enhance mitochondrial respiration. **A** The significantly enriched pathways (*P* < 0.05) in the Reactome pathway analysis of the selected mRNAs according to inosine vs. -G-Q treatment. **B** Western blots showing the puromycin labeled global nascent proteins level in MDA-MB-231 cells with indicated treatment. **C** Schematic diagram of OPP labeling for detecting the nascent proteins. **D** Venn diagram shows overlapping nascent proteins enriched in both inosine treatment and glucose treatment compared with -G-Q treatment. **E** The significantly enriched pathways (*P* < 0.05) in the KEGG pathway analysis of the overlapping nascent proteins enriched from the treatment above. **F** The level of nascent proteins labeled with OPP in carbon metabolism and results are shown as a heat map. **G** MDA-MB-231 cells were treated with indicated above, and OCR was determined by extracellular flux analysis. A representative plot of OCR over time with the addition of oligomycin (0.5 μM), mitochondrial uncoupler p-trifluoromethoxy carbonyl cyanide phenyl hydrazine (FCCP) (1 μM), and electron transport inhibitors rotenone and antimycin (0.5 μM), as indicated (two-way ANOVA, *n* = 3 biological replicates). **H** Abundance of metabolites during oxidative phosphorylation in MDA-MB-231 cells with -G-Q, inosine, and glucose treatment (one-way ANOVA, *n* = 3 biological replicates). **I** Representative IHC images showing indicated protein staining of core region tissues separated from 4T1 tumors. Scale bar, 50 μm. **(J)** Western blots showing the nascent protein levels OPP labeled in MDA-MB-231 cells (Left) and 4T1 cells (Right) with indicated treatment. **K** Up: MDA-MB-231 cells labeled with Lck-GFP (231/Lck-GFP) were treated with CS inhibitor (4 mM l-Tyrosine) or 231/Lck-GFP/ACO1 KD cells in glucose–glutamine-deficient medium supplemented with inosine. The fluorescent images were taken at the indicated time. Scale bar, 1000 μm. Bottom: Quantification of the number fold change of cells (one-way ANOVA, *n* = 3 biological replicates).

Translation, as one of the downstream effects of the mTORC1 signaling pathway, was identified as significantly enriched upon inosine treatment compared with starvation (Fig. 4A and S4A). To determine whether inosine-mediated mTORC1 activation was sufficient to influence protein synthesis, we used the surface sensing of translation (SUnSET) technique [30], which took advantage of puromycin acting as a mimetic for tyrosine during mRNA translation. Induced incorporation of puromycin into newly synthesized proteins was detected after inosine feeding, suggesting significantly nascent protein synthesis in cancer cells under inosine treatment (Fig. 4B). To directly visualize the translation, we performed an O-propargyl-puromycin (OPP)-mediated pulldown assay to detect nascent synthesized proteins under inosine treatment (Fig. 4C). Further mass spectrometry showing around 268 nascent proteins were overlappingly enriched in both inosine and glucose treatment when compared to starvation group (Fig. 4D). Intriguingly, both carbon metabolism and citrate cycle-related pathways were significantly enriched (Fig. 4E), especially enzymes residing in the TCA cycle (Fig. 4F). Indeed, inosine treatment upregulated the oxygen consumption rate (OCR) of cancer cells under starvation (Fig. 4G). LC–MS/MS assay also confirmed an elevated abundance of iso-citric acid, one of the TCA cycles intermediates, during inosine-treated starved cancer cells (Fig. 4H), rather than glycolysis (Fig. S4B). However, we found palmitic acid and linoleic acid were significantly reduced under inosine treatment, suggesting the oxidation process might use them as an alternative fuel (Fig. S4C). Additional EM images confirmed an increased mitochondria content upon inosine treatment (Fig. S4D, E).

To elucidate the molecular mechanism, we further examined the expression of nascent TCA cycle enzymes by western blots and found CS and ACO1 were significantly upregulated with merely altered total protein abundances (Fig. 4I, J). To further examine which enzyme was involved in the inosine-mediated survival, we knocked down ACO1 and inhibited CS activity with l-Tyrosine in MDA-MB-231 cells, respectively (Fig. S4F). Inosine failed to rescue the survival rate of 231/ACO1 KD cells and l-tyrosine-treated MDA-MB-231 cells under starvation (Fig. 4K and S4G). Intriguingly, when we compared the pool of inosine-induced nascent proteins and the mitochondrial proteins enriched in TC-derived extracellular vesicles (EVs), we found there were three proteins, ACAA2, COX6C, ATP5F1, were enriched in TC-derived EVs than TM derived EVs (Fig. S4H, I), suggesting inosine might contribute the secretion of the nascent protein through EVs. Conclusively, these data indicated inosine, as a cellular signaling metabolite, stimulated the mTORC1 signaling pathway and mitochondrial respiration by inducing nascent TCA enzyme expression.

### The regulation of inosine of mitochondrial metabolism widely exists in BC tumors

**Fig. 5:**
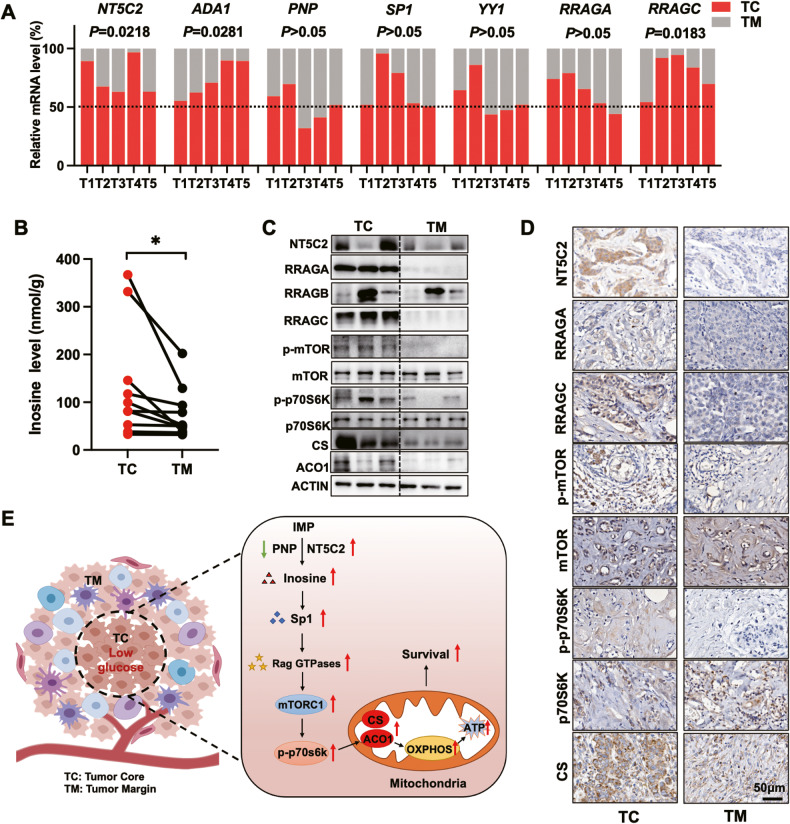
Inosine-mediated mitochondrial metabolism widely exists in BC patients. **A** Relative RNA levels in core and margin regions tissues from BRCA tumors were determined by RT-qPCR (paired two-tailed Student’s *t*-test, *n* = 5 per group). **B** Tissues from core and margin regions of BRCA tumors used for inosine detection. Inosine concentrations were measured by ELISA kit, and results were standardized with protein concentration (paired two-tailed Student’s *t*-test, *n* = 10 per group). **C** Western blots showing the protein expression levels in the core and margin regions of tissues separated from BRCA tumors. **D** Representative IHC images showing indicated protein staining of core and margin region of tissues separated from BRCA tumors. Scale bar, 50 μm. **E** Schematic summary of this study.

To determine whether the inosine-mediated mitochondrial metabolism widely existed in patient samples, we examined a cluster of BC patients’ tumors and found there were more *NT5C2*, *ADA1*, and *RRAGC* expression but nearly no alteration of *PNP*, *RRAGA*, and *SP1* in TC than that in TM (Fig. 5A). However, TCGA analysis showed both higher expression of ADA1 and SP1 in BC tumors than normal tissues (Fig. S5A, B). Of note, a higher level of SP1 resulted in a lower survival rate (Fig. S5C). Simultaneously, more inosine was detected in TC regions of patient tumors (Fig. 5B). Both NT5C2 and SP1 were highly correlated with the expression of Rag GTPases. NT5C2 expression was highly positively correlated with RRAGC and RRAGD (Fig. S5D, E). SP1 was highly positive correlated with RRAGA, RRAGB, and RRAGC (Fig. S4F–H). Indeed, there were more NT5C2, SP1, RRAGA, RRAGC, p-p70S6K, and p-mTOR expressions in TC when compared to TM (Fig. 5C, D). Interestingly, along with CS and ACO1, other TCA enzymes, such as MDH2 and SDHB, were also elevated (Fig. S5I). However, there was less PNP expression in TC when compared to TM (Fig. S5I). In summary, in our depicted model, we identified under starvation, inosine induced SP1/Rag GTPases axis to activate mTORC1, thereby inducing nascent CS and ACO1 expression to further promote mitochondrial respiration (Fig. 5E).

## Discussion

Inosine has been reported as an alternative fuel to provide ATP and biosynthetic precursors for cancer cells or T cells under starvation. The effector T cells metabolize inosine into hypoxanthine and phosphorylated ribose under glucose starvation, enhancing the anti-tumor efficacy of immune checkpoint blockade [31]. However, our finding provides a distinct mechanism that cancer cells could utilize inosine to support cell survival in the absence of glucose through the induction of Rag GTPase levels and the synthesis of nascent metabolic enzymes in the TCA cycle, which extends the function of inosine as a signal transmitter. In our study, inosine upregulates the Rag GTPases by inducing the expression of transcription factor SP1. Nutrient starvation mediated the glycosylation of SP1 is also associated with altered proteasome susceptibility [32]. Although protein synthesis is one of the most energy-consuming processes in the cells, the nascent proteins induced by inosine might play an essential role in the regulation of cell survival. Indeed, previous reports showed mTORC1 stimulates the synthesis of nucleus-encoded mitochondria-related proteins, such as TFAM, mitochondrial ribosomal proteins, and components of complexes I and V [33, 34]. Most of the nascent proteins we identified are TCA cycle-related enzymes, suggesting cancer cells enhance catabolism for energy production to maintain survival under starvation. Partial nascent proteins might secrete in the form of EVs and play systemic regulation. Additional TCA cycle intermediates such as iso-citrate identified in our study might be essential for cell survival, as mitochondrial TCA cycle metabolites have been reported to control physiology and disease and interplay with mTORC1 [35, 36]. TC region has the highest abundance of cancer cells in solid tumors [37]. Extreme environments caused by glucose deprivation and hypoxia force cancer cells to utilize extra carbon-based compounds such as lactate, acetate, and nucleotides, including inosine, as fuel to meet the energy requirement for rapid proliferation [38–40]. Inosine metabolism has been rewired in cancer cells, and whether inosine utilizes a similar regulatory strategy to relieve tumor-imposed metabolic restrictions in immune cells (such as T cells) is still waiting for future investigation.

Inosine is emerging as a highly versatile bioactive compound and second messenger of signal transduction in cells with diverse functional abilities in different physiological and pathological states [41]. Several researchers found that inosine interacts with cell surface purinergic receptors directly to play a signal transduction function or releases ribose moiety to generate energy [31, 42]. However, we did not observe significant downregulation of the mTORC1 signaling pathway after blocking PNP/ADORA1&ADORA2A, although causing a little bit of reduction of cell viability than the control groups. The concrete mechanism needs further exploration.

As a signal, inosine connects mitochondrial respiration and nutrient starvation in cancer cells by stimulating the mTORC1 signaling pathway. Indeed, mTOR, as a central coordinator of amino acid availability and allocation, has been evidenced by its contribution to mitochondria regulation [43]. For instance, mTOR promotes the replication of mitochondrial DNA and the expression and activities of enzymes residing in mitochondria, thus affecting mitochondrial oxidative phosphorylation [44]. Another report shows mTORC1 stimulates the translation of mitochondrial fission process 1 (MTFP1) to control mitochondrial fission [45]. Moreover, the mTOR also regulates the synthesis of mitochondrial regulatory factors by regulating the translation process of mRNAs, thus affecting mitochondrial biosynthesis and respiration [34]. In addition to regulating the translation of nuclear-encoded mitochondria-related mRNAs, mTOR also regulates the transcription factors for metabolism or mitochondrial genes, such as yin-yang 1 (*YY1*) and hypoxia-inducing factor-1α (*HIF-1α*) [29, 46]. Although all the literature highlights the connection between mTOR and mitochondria and mRNA translation, the influences of inosine on the regulation of apoptosis and autophagy under nutrient starvation still need further investigation.

Inosine has also been reported with an essential effect on thermogenic anti-inflammation and sustainment cell survival under glucose starvation [42, 47–49]. Single nucleotide plays a vital role in cell progress, such as energy metabolism based on nucleotide cofactors, post-transcriptional modification with A-to-I RNA editing, cell cycle, and immune evasion [50–52]. The accumulated inosine in our study derives from IMP either from the salvage pathway or de novo nucleotide biosynthesis. Although de novo nucleotide biosynthesis is considered as an energy-intensive process, which is highly dependent on carbon and nitrogen sources and requires multiple inputs distributed between multiple pathways and organelles, especially mitochondria [53]. Inosine is beneficial for cancer cells, especially in providing energy under glucose starvation. However, a key metabolic hallmark of increasing nucleotide de novo synthesis to fit cell growth is universal reprogramming in cancer cells [54]. IMP, a central intermediate metabolite in the purine metabolism pathway, is the precursor to generate adenosine monophosphate (AMP), inosine, and guanosine monophosphate (GMP) [55]. Up to date, a quantity of anti-cancer drugs, most purine nucleotide analogs, including 6-mercaptopurine, 6-thiocitrulline, and methotrexate which target the key enzymes in purine metabolism in clinical [56]. CRCD2, a first-in-class specific small molecule inhibitor of the cytosolic 5′ nucleotidase II (NT5C2), has been reported to be broadly active against NT5C2 in acute lymphoblastic leukemia (ALL) [57]. Whether any NT5C2 inhibitor, such as CRCD2 or PNP agonizts, can effectively block inosine accumulation is awaiting further investigation. Overall, targeting inosine metabolism might be a promising strategy for cancer treatment.

## Materials and methods

### Cells and constructs

Cell lines applied in this study were obtained from American Type Culture Collection including MDA-MB-231 (HTB-26), 4T1 (CRL-2539), 293 T (CRL-3216), SW480 (CCL-228), Hep3B (HB-8064) and A549 (CCL-185) were cultured in DMEM (Gibco) containing 10% FBS and 1% penicillin/streptomycin. All cell lines are authenticated and free from mycoplasma contamination. Both glucose and glutamine-free medium was purchased from Thermo Fisher (A1443001), supplemented with 16.67 mM inosine (Sigma-Aldrich, 58-63-9), 3 g/L glucose (BioFroxx, 50-99-7) with or without dialyzed FBS (Gibco, 26400044). All cell lines were cultured in an atmosphere of 5% CO_2_ incubator at 37 °C. MDA-MB-231/Lck-GFP cells were constructed by transfection of Lck-GFP expression plasmid (Addgene, #61099) into MDA-MB-231 and followed by selection in G418 and sorting of GFP^+^ cells by Flow Cytometry. MDA-MB-231/SP1 KD cells were constructed using short hairpin RNA (shRNA) targeting SP1 sequence (shSP1-1:CCACTCCTTCAGCCCTTATTA and shSP1-2: GCAGGATGGTTCTGGTCAAAT) and the corresponding oligonucleotides were cloned into pLKO.1 puro vector (Addgene, #8453). Cells were transduced by the lentivirus and selected in puromycin. A similar strategy was used to construct MDA-MB-231/ACO1 KD cells using shRNAs (DNA sequences GGAATGTTTCGAGATTTCA), MDA-MB-231/PNP KD cell line (DNA sequences GCTCTCAGTACCTGGAAACAA), MDA-MB-231/ ADORA1 KD cell line (DNA sequences CCCAGCATCCTTACCTACATT), MDA-MB-231/ADORA2A KD cell line (DNA sequences CCACACCAATTCGGTTGTGAA), 4T1/NT5C2 KD cell line(DNA sequences CGGATTAAGAAAGTAACTCAT), 4T1/SP1 KD cell line (DNA sequences CCTTCACAACTCAAGCTATTT), 4T1/RRAGA KD cell line (DNA sequences GAGCTACGTTCTTGGTGATTT), 4T1/RRAGB KD cell line (DNA sequences GCAACAATACTAGACCGTATA), and 4T1/RRAGC KD cell line (DNA sequences GCATCTATGACCATTCAATAT). To construct MDA-MB-231/SP1 overexpression cells, forward primer 5′-GCCACCATGAGCGACCAAGATCACTCCATGG and reverse primer 5′-TCAGAAGCCATTGCCACTGATATTA were used to clone the sequence of human *SP1*.

### Antibody

Antibodies against p70S6K (sc-8418), LAMP1 (sc-19992), and phospho-mTOR (sc-293133) were purchased from Santa Cruz Biotechnology (USA). Antibodies against phosphor-p70S6K (AP0564), RRAGA (A15134), RRAGD (A9979), and ADORA1 (A5219), ADORA2A (A1587) were purchased from ABclonal (China). Antibodies against NT5C2 (15223-1-AP), RRAGB (13023-1-AP), RRAGC (26989-1-AP), SP1 (21962-1-AP), YY1 (66281-1-lg), mTOR (66888-1-Ig), Actin (66009-1-lg), GAPDH (10494-1-AP), ADA1 (13328-1-AP), MDH2 (15462-1-AP), MDH1 (15904-1-AP), ACO1 (12406-1-AP), CS (16131-1-AP), GLUD1 (14299-1-AP), and SDHB (10620-1-AP) were purchased from Proteintech (China). Antibody against PNP (DF8260) was purchased from Affinity (China). Antibody against puromycin (MABE343) was purchased from Sigma-Aldrich (USA). Antibody against Ki67 (ab15580) was purchased from Abcam (UK).

### Western Blot analysis

Cells or tissues were homogenized in lysis buffer containing 50 mM Tris (pH 7.4), 500 mM NaCl, 1% NP40, 20% glycerol, 5 mM EDTA and 1 mM PMSF, 1 mM DTT (Roche), supplemented with protease and phosphatase inhibitor cocktails (Roche). Proteins were separated by electrophoresis on a 10% or 12% SDS polyacrylamide gel. Protein detection was performed using the antibodies described above.

### RNA isolation and RT-qPCR

Total RNA was harvested from cultured cells or tissues using the TRIzol reagent (Invitrogen, 15596026), followed by cDNA synthesis using the MonScript RTIII Super Mix with dsDNase kit (Monad Biotech, MR05201). A CFX Connect real-time PCR system (Bio-Rad Laboratories) was used to perform quantitative real-time PCR (RT-qPCR) of cDNA samples using MonAmp ChemoHS qPCR Mix (Monad Biotech, MQ00401). Totally, 200 μL of chloroform was added, gently mixed, and incubated for 10 min on ice. After incubation, microcentrifuge tubes were centrifuged for 10 min at 12,000×*g* at 4 °C. The upper aqueous phase was transferred to new tubes, and an equal volume of isopropanol was added and mixed with incubation at −20 °C overnight. Samples were centrifuged at 12,000×*g* for 15 min at 4 °C. Aspirated off liquid and added 500 μL of cold 75% ethanol to wash the pellet. The RNA pellet was air-dried for a few minutes at room temperature and subsequently resuspended in 20 μL of RNase-free water. All the other primers are shown in Supplementary Table 1.

### Real-time fluorogenic

Cells (MDA-MB-231/Lck-GFP and MDA-MB-231/Lck-GFP/ACO1 KD) with or without 4 mM l-tyrosine treatment, were plated to the 12-well microplates and cultured with DMEM medium with normal glucose level for 24 h, following the replacement of glucose and glutamine-free medium with additional inosine or glucose supplementary. The dynamic fluorescent images were captured by Cytation Multi-Mode Reader (BioTek, USA).

### CCK8 assay

Totally, 1 × 10^4^ of MDA-MB-231 cells were plated into the 96-well microplates and incubated with 100 μL of DMEM medium with normal glucose level per well for 24 h. Cells were replaced the next day with the indicated conditional medium and an additional 10 μL of CCK8 reagent (Yeasen Biotechnology, 40203ES88) for another 2 h-culture. The OD450 values were captured by Epoch (BioTek, USA) at each indicated time point.

### Respiration assays

The OCR of MDA-MB-231 cells treated with glucose–glutamine-free medium (-G-Q) or supplemented with inosine or glucose was determined using an XFe96 Extracellular Flux Analyzer (Agilent). The day before the assay, the Seahorse cartridge (Agilent, 102601-100) was placed in the XF calibrant (Agilent, 100840-000) and incubated overnight at 37 °C. Around 8 × 10^3^ of MDA-MB-231 cells per well were initially plated in XF96 microplates (Agilent, 101085-004) and treated with the above three media for 12 h, then the culture medium was changed to XF assay medium (Agilent, 103575-100) supplemented with 2 mM glutamine (Agilent, 103579-100), 10 mM glucose (Agilent, 103577-100), and 1 mM pyruvate (Agilent, 103578-100) for OCR. Cells were incubated in a CO_2_-free incubator for 1 h at 37 °C to allow for temperature and pH equilibration prior to loading into the XFe96 analyzer for measurement following the manufacturer’s protocol. Basal respiration was derived by subtracting the third OCR reading following antimycin A addition from the third basal OCR reading. Uncoupled and maximal OCR was determined using Oligomycin (0.5 μM) and FCCP (1 μM) (Agilent, 103015-100), respectively. Complex I-dependent respiration was inhibited with Rotenone and antimycin (0.5 μM) (Agilent, 103015-100).

### Immunofluorescence staining and confocal microscopy

Cultured MDA-MB-231 cells were seeded on collagen-coated coverglass and stained for mTOR and marker proteins LAMP1 of lysosomes. Briefly, cells were fixed with 4% formaldehyde and then permeabilized with 0.2% Triton X-100. Primary antibodies were diluted with PBS and incubated overnight at 4 °C, followed by appropriate secondary antibodies at room temperature for 1 h. Nuclei were then counterstained with DAPI and sealed plate.

### Histology and immunostaining

The 5 μm-thick tumor sections from human and mouse tumor tissue were transferred to adhesive slides and maintained in a drying oven at 60 °C for 2 h. Tumor sections were deparaffinized and rehydrated. The sections were immersed in citrate solution and boiled at high temperatures for antigen repair. After cooling to room temperature, blocked with endogenous peroxidase for 10 min. After 3 times of washing with PBS, 3% BSA was used to block for 30 min. The primary antibody was incubated at 4 °C overnight. After washing with PBS for 3 times, the secondary antibody was incubated at room temperature for 1 h. Subsequently, the tissue sections were incubated with a DAB kit (PV-9000) at 37 °C for 2 min and counterstained with Harris’s hematoxylin for 10 s. For brightfield images, slides were scanned by Aperio versa slide scanner (Leica), and images were obtained with Aperio ImageScope software (Leica).

### Isolation and proteomic characterization of TC and margin tissue-derived EVs

A small (~50 mg) piece of tissue was weighed and briefly sliced on dry ice and then incubated in 100 U/mL collagenase type I in HANKS solution at 37 °C. The dissociated tissue was spun at 300×*g* for 10 min at 4 °C. The supernatant was transferred to a new tube and centrifuged at 2000×*g* for 15 min at 4 °C. Cell-free supernatant was filtered through the 0.22 μm filter gently and slowly for further removal of cell debris and spun at 10,000×*g* for 30 min at 4 °C. EVs were pelleted by ultracentrifugation at 110,000×*g* for 70 min, and resuspended in PBS. Added appropriate amount of protein lysate (8 M urea, 1% SDS), with protease inhibitor to inhibit protease activity. The mixture was treated by ultrasound for 2 min at a low temperature, following splitting for 30 min. After centrifugation at 12,000×*g* at 4 °C for 30 min, the concentration of protein supernatant was determined by the Bicinchoninic acid (BCA) method by Pierce BCA Protein Assay Kit (Thermo Fisher, USA). Protein quantification was performed according to the kit protocol. Added TEAB (Triethylammonium bicarbonate buffer) into 100 μg protein samples to the final TEAB concentration of 100 mM. Then added, TCEP (tris (2-carboxyethyl) phosphine) to the final concentration of 10 mM and reacted for 60 min at 37 °C. Following, add IAM (Iodoacetamide) to the final concentration of 40 mM and react for 40 min at room temperature under dark conditions. Added a certain percentage (acetone: sample v/v = 6:1) of pre-cooled acetone to each sample and settled for 4 h at −20 °C. After centrifugal for 20 min at 10,000×*g*, the sediment was collected and add 100 µL 100 mM TEAB solution to dissolve. Finally, the mixture was digested with trypsin overnight at 37 °C added at a 1:50 trypsin-to-protein mass ratio. The peptides were vacuum dried and then resuspended with 0.1% TFA. Samples were desalted with HLB and vacuum dried. Peptide concentrations were determined by peptide quantification kit (Thermo Fisher, Cat#23275). Loading buffer was added to each tube to prepare samples for mass spectrometry analysis, and the concentration of each sample was 0.25 µg/µL. Trypsin-digested peptides were analyzed by an EASY nLC-1200 system (Thermo Fisher, USA) coupled with a timsTOF Pro2 (Bruker, Germany) mass spectrometer at Majorbio Bio-Pharm Technology Co. Ltd. (Shanghai, China). Briefly, the C18-reversed phase column (75 μm × 25 cm, Ionopticks, USA) was equilibrated with solvent A (A:2% ACN with 0.1% formic acid) and solvent B (B: 80% ACN with 0.1% formic acid). The peptides were eluted using the following gradient: 0–45 min, 3–28% B; 45–50 min, 28–44% B; 50–55 min, 44–90% B; 55–60 min, 90–90% B. The tryptic peptides were separated at a flow rate of 250 nL/min. Peptides were separated by an ultrahigh performance liquid phase system subjected to a capillary ion source and then analyzed by timsTOF Pro2 (Bruker, Germany), and the electrospray voltage was 1.5 kV. The peptide parent ions and their secondary fragments were detected and analyzed using high-resolution TOF. The secondary MS scanning range was 100–1700 m/z. Data acquisition on the timsTOF Pro2 was collected using the parallel accumulation serial fragmentation (PASEF) acquisition mode. After the first MS stage, the second MS stage (charge number of the parent ions was 0–5) was recorded using the 10 PASEF mode. A dynamic exclusion time of 24 s was used for the MS/MS scan.

### Mass spectrometry imaging

Frozen tissue samples were fixed in three drops of distilled water during the cutting stage. The tissues were sectioned at 12 μm thickness using a Leica CM1950 cryostat (Leica Microsystems GmbH, Wetzlar, Germany) at −20 °C. Afterward, the tissue sections were placed in groups on electrically conductive slides coated with indium tin oxide (ITO), and the slides with tissue sections were dried in a vacuum desiccator for 30 min. Desiccated tissue sections mounted on ITO glass slides were sprayed using an HTX TM sprayer (Bruker Daltonics, Germany) with 15 mg/mL DHB (2,5-dihydroxybenzoic acid), dissolved in 90%:10% Acetonitrile:water. The sprayer temperature was set to 60 °C, with a flow rate of 0.12 mL/min pressure of 5 psi. Twenty-six passes of the matrix were applied to slides with 5 s of drying time between each pass. MALDI timsTOF MSI experiments were performed on a prototype Bruker timsTOF flex MS system (Bruker Daltonics, Bremen, Germany) equipped with a 10 kHz smart beam 3D laser. Laser power was set to 60% and then fixed throughout the whole experiment. The mass spectra were acquired in positive mode. The mass spectra data were acquired over a mass range from m/z 50–1300 Da. The imaging spatial resolution was set to 50 μm for the tissue, and each spectrum consisted of 400 laser shots. MALDI mass spectra were normalized with the Root Mean Square, and the signal intensity in each image was shown as the normalized intensity. MS/MS fragmentations performed on the timsTOF flex MS system in the MS/MS mode were used for further detailed structural confirmation of the identified metabolites. All the metabolites are shown in Supplementary Table 2.

### The LC-MS/MS detected the nascent protein by OPP labeling

MDA-MB-231 and 4T1 cells were cultured with glucose/glutamine-deficient medium or supplemented with inosine or glucose for 8 h, then added 20 μM OPP (Cat#1407-5, Click Chemistry Tools) to the medium for further culturing 2 h. Subsequently, cells were collected to extract the proteins on ice. Totally, 250 µg proteins were combined with biotin by click chemistry, allowing biotin picolyl azide (Cat#1167-5, Click Chemistry Tools) and Click-&-Go® Protein Reaction Buffer Kit (Cat#1262, Click Chemistry Tools) from Click Chemistry Tools following the provided instructions. After that, methanol and chloroform were added to the mixture to precipitate proteins, then washed with methanol. The pellets were resuspended in PBS with 1% SDS and measured protein concentration by BCA assay after air drying. Ten micrograms of protein were stored at −80 °C as input control. Totally, 150 µg of proteins were incubated with High Capacity Streptavidin Magnetic Beads (Cat#1497-1, Click Chemistry Tools) at 4 °C overnight with slow rotation. Global nascent proteins were dissolution with 2× loading buffer in the supernatant fluid after beads elution. The proteins were prepared for LC/MS analysis by SDS-PAGE electrophoresis. The nascent proteins are shown in Supplementary Table 3.

### Puromycin labeling for detection of global nascent protein synthesis

Cells were added with 10 µM puromycin and treated for 1 h after glucose–glutamine-deficient medium or supplemented with inosine or glucose cultured for 8 h. Then cells were washed 3 times and collected cell scraper. All proteins were extracted by RIPA with protease inhibitor. The specimen was prepared for SDS-PAGE electrophoresis after boiling with a loading buffer. Puromycin antibody was 1:5000 dilution.

### Measurements of metabolites levels

Inosine levels in cells and in tissues were measured with an ELISA kit (Sigma-Aldrich, 58-63-9) according to the manufacturer’s instructions. The final concentration was normalized to standard volume or protein mass.

### ATP detection

Cells and xenografted TC and margin regions were separated and cracked using a lysis solution in the ATP detection kit (S0027, Beyotime). All the samples were prepared for further detection according to the manufacturer’s instructions. The final concentration was normalized to protein mass.

### Crystal violet staining

Totally, 1 × 10^5^ of cells were plated in 6-well microplates for survival analysis the day before staining. The next day cells were replaced with the indicated conditional medium and cultured for 8 h. Cells were washed with PBS for 3 times, then added 0.1% crystal violet solution staining for 30 min, and finally washed with water, and images were generated using a scanner (EPSON Scan).

### Determination of metabolic composition

Transferred all cell samples into a 2 mL centrifuge tube and added 100 mg glass bead. Then added, 1000 μL acetonitrile (ACN): methanol: H_2_O mixed solution (2:2:1, V / V / V) (stored at 4 °C), vortexed for 30 s. Put the centrifuge tube containing the sample into the 2 mL adapter matched with the instrument, immerse it in liquid nitrogen for rapid freezing for 5 min, take out the centrifuge tube, and thaw at room temperature; put the centrifuge tube into the 2 mL adapter again, installed it into the tissue grinder and ground it at 55 Hz for 2 min. Repeated the preceding steps two times. Then took out the centrifuge tube, centrifuged it for 10 min at 12,000 rpm and 4 °C, took all the supernatant, transferred it to a new 2 mL centrifuge tube, concentrated, and dried it. Accurately added 300 μL acetonitrile: 2-Amino-3-(2-chloro-phenyl)-propionic acid (4 ppm) solution prepared with 0.1% formic acid (1:9, V / V) to redissolve the sample, filtered the supernatant by 0.22 μm membrane and transferred into the detection bottle for LC–MS detection. The LC analysis was performed on a Vanquish UHPLC System (Thermo Fisher Scientific, USA). Chromatography was carried out with an ACQUITY UPLC ® HSS T3 (150 × 2.1 mm, 1.8 µm) (Waters, Milford, MA, USA). The column was maintained at 40 °C. The flow rate and injection volume were set at 0.25 mL/min and 2 μL, respectively. For LC-ESI ( + )-MS analysis, the mobile phases consisted of (C) 0.1% formic acid in acetonitrile (v/v) and (D) 0.1% formic acid in water (v/v). Separation was conducted under the following gradient: 0 ~ 1 min, 2% C; 1 ~ 9 min, 2% ~ 50% C; 9 ~ 12 min, 50–98% C; 12–13.5 min, 98% C; 13.5–14 min, 98–2% C; 14–20 min, 2% C. For LC–ESI (−)-MS analysis, the analytes were carried out with (A) acetonitrile and (B) ammonium formate (5 mM). Separation was conducted under the following gradient: 0–1 min, 2% A; 1–9 min, 2–50% A; 9–12 min, 50–98% A; 12–13.5 min, 98% A; 13.5–14 min, 98–2% A; 14–17 min, 2% A [58]. Mass spectrometric detection of metabolites was performed on Q Exactive (Thermo Fisher Scientific, USA) with an ESI ion source. Simultaneous MS1 and MS/MS (Full MS-ddMS2 mode, data-dependent MS/MS) acquisition was used. The parameters were as follows: sheath gas pressure, 30 arb; aux gas flow, 10 arb; spray voltage, 3.50 kV and −2.50 kV for ESI (+) and ESI (−), respectively; capillary temperature, 325 °C; MS1 range, m/z 81–1000; MS1 resolving power, 70,000 FWHM; number of data dependant scans per cycle, 10; MS/MS resolving power, 17500 FWHM; normalized collision energy, 30%; dynamic exclusion time, automatic [59]. The metabolic composition was shown in Supplementary Table 4.

### Electron microscope

MDA-MB-231 and 4T1 cells were excised and fixed with 4% formaldehyde in 100 mM phosphate buffer (PB, pH = 7.4) and 2.5% glutaraldehyde for 1 h after culturing with the above three treatments. Then collected cell deposits and gradient centrifugation. Samples were postfixed with 2% osmium tetroxide (OsO_4_) for 1 h on ice, then stained with aqueous uranyl acetate at 4 °C overnight, protected from light. Samples were dehydrated with graded series of cold ethanol (30, 50, 70, 80, 95, and 100%) the next day. After being washed three times in epoxypropane, the samples were infiltrated sequentially in 1:1 (v:v) epoxypropane/epoxy resin (4 h), 1:2 (v:v) epoxypropane/epoxy resin (overnight), 100% fresh epoxy resin (9 h), and finally 100% fresh epoxy resin (48 h) at 60 °C for polymerization. The thin sections were placed onto carbon-coated copper grids and stained with aqueous uranyl acetate and lead citrate, and observed using a JEM-1400 plus electron microscope operated at 100 kV.

### RNA sequencing and analysis

RNA sequencing was performed by Majorbio (Shanghai, China), and the transcriptome library was prepared following TruSeq RNA sample preparation Kit from Illumina (San Diego, CA) using 1 μg of total RNA. Libraries were size selected for cDNA target fragments of 300 bp through 2% Low Range Ultra Agarose followed by PCR amplified using Phusion DNA polymerase (NEB) for 15 PCR cycles. After quantified by TBS380, the paired-end RNA-seq sequencing library was sequenced with the Illumina HiSeq xten/NovaSeq 6000 sequencer (2 × 150 bp read length). The raw paired-end reads were trimmed and quality controlled by SeqPrep (https://github.com/jstjohn/SeqPrep) and Sickle (https://github.com/najoshi/sickle) with default parameters. Then clean reads were separately aligned to the reference genome with orientation mode using HISAT2 (https://daehwankimlab.github.io/hisat2) software. The mapped reads of each sample were assembled by StringTie (https://ccb.jhu.edu/software/stringtie/index.shtml?%20t=example) in a reference-based approach. The expression level of each transcript was calculated according to the transcripts per million reads (TPM) method. RSEM (http://deweylab.biostat.wisc.edu/rsem/) was used to quantify gene abundances. Eventually, differential expression analysis was performed using the DESeq2.

### Mice

Animal experiments in this study were approved by and performed in accordance with the institutional animal care and use committee (IACUC) at the College of Life Science, Wuhan University. Six-to-eight weeks old female NOD/SCID/IL2Rγ-null (NSG) mice were purchased from Shanghai Model Organisms Center (for MDA-MB-231 xenografted model), or female BALB/c mice were purchased from the Center for Disease Control (CDC; Hubei, China) (for 4T1 xenografted model) were used in this study. Mice were maintained under specific pathogen-free conditions and housed four to five mice per cage at a 12 h light/dark cycle at a relative humidity of 30–70% and room temperature of 22.2 ± 1.1 °C and were allowed free access to food and water. MDA-MB-231 xenografted models were established by injecting orthotopically 2 × 10^5^ cells with an equal volume of Matrigel (Corning, 356234) into the fourth pair of mammary fat pads. 4T1 and 4T1/NT5C2 KD xenografted models were established by injecting 3 × 10^5^ cells. 4T1/SP1 KD and 4T1/Rag GTPases KD xenografted models were established by injecting 1 × 10^6^ cells, 4T1 WT as control. For Plicamycin treatment, after 4T1 tumor formation, the tumor-bearing mice were randomly divided into two groups. Plicamycin solution (1.5 mg/kg) was injected into the region of TC every other day at 50 μL per tumor, and normal saline was used as a control. For inosine injection into the 4T1 tumor, inosine solution (1.5 g/L) was injected into the region of TC every other day at 100 μL per tumor after three weeks and was administered seven times over a 14-day period, and normal saline was used as a control. Tumor volume was calculated using the formula (length × width^2^)/2. At the beginning of each experiment, mice were randomly assigned to each group. For lung, liver, and colon cancer models, 1 × 10^6^ of A549 cells, Hep3B cells, and SW480 cells suspended in 100 μL PBS were injected subcutaneously into the right flank of nude mice. When the volumes of xenografted tumors reached an average of 1200 mm^3^, mice were sacrificed and collected for subsequent analysis.

### Human BC specimens

Archived samples from patients with BC applied in this study were collected in accordance with the Clinical Research Ethical Committee of Renmin Hospital of Wuhan University. All participants provided written informed consent. Unstained paraffin-embedded tissue sections and fresh tissues of BC patients were obtained by voluntary patient consent from Renmin Hospital of Wuhan University. All the patient information is shown in Supplementary Table 6.

### Software

Equipment build-in software was used for data collection, including the CFX Manager Software version 2.0 for real-time PCR data; Prism 9 and R Studio 1.1.463 was used for most data analysis and statistics; GSEA 4.2.2 was used for gene set enrichment analysis of RNA-seq data; Metascape (https://metascape.org/) was used for Kyoto Encyclopedia of Genes and Genomes (KEGG) enrichment analysis of secreted protein mass spectrum. The websites (https://jaspar.genereg.net/ and http://bioinfo.life.hust.edu.cn/HumanTFDB#!/) were used to predict transcription factors. This study used the GTBA database to explore the relationship between genes (https://guotosky.vip:13838/GTBA/). Gene Expression Profiling Interactive Analysis (GEPIA) was used for gene expression analysis based on tumor and normal samples, and the survival curve was from the Cancer Genome Atlas (TCGA) and the Genotype-Tissue Expression (GTEx) databases.

### Statistics and reproducibility

All quantitative data were presented as mean ± standard deviation. Two-tailed Student’s *t*-tests were used for the comparison of means of data between the two groups. For multiple independent groups, one-way with post hoc Tukey tests or two-way ANOVA with Šídák’s tests were used. Values of *P* < 0.05 were considered significant. The sample size was generally chosen based on preliminary data indicating the variance within each group and the differences between groups. Pearson correlation analysis was employed for analyzing the strength and direction of the linear relationship between two variables and represented as *R*-value. All samples received the proper procedures with confidence and were included in analyses. Animals were randomized before treatments. Western blots were repeated independently three times with similar results, and representative images are shown.

## Supplementary information

Original Data File

Supplementary figures and tables

## Data Availability

The RNA-seq generated in this study has been deposited in the NCBI Gene Expression Omnibus (GEO) with accession code GSE225643 (starved MDA-MB-231 cells with additional inosine or glucose treatment) and accession code GSE222291 (tumor core and margin). The predśicted transcription factor can be found in Supplementary Table 5. All other data supporting the findings of this study are available from the corresponding author upon reasonable request.
