# Supplementary figures and images for: Inosine enhances tumor mitochondrial respiration by inducing Rag GTPases and nascent protein synthesis under nutrient starvation
Source: Cell Death Dis. 2023 Aug 2;14(8):492. doi: 10.1038/s41419-023-06017-2 (PMC10397262; doi:10.1038/s41419-023-06017-2)

**Figure 1**

**1G**

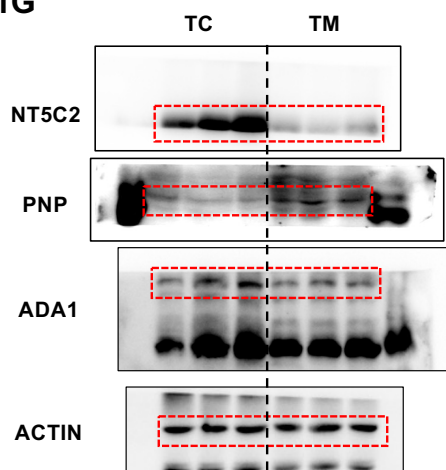

**Figure S1**

**S1F**

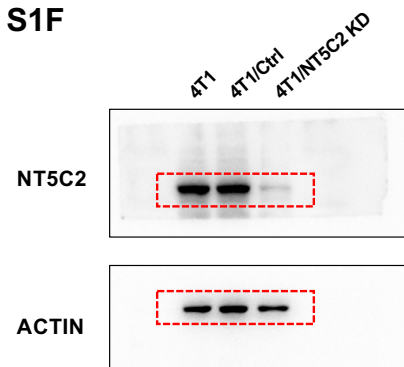

Figure 2

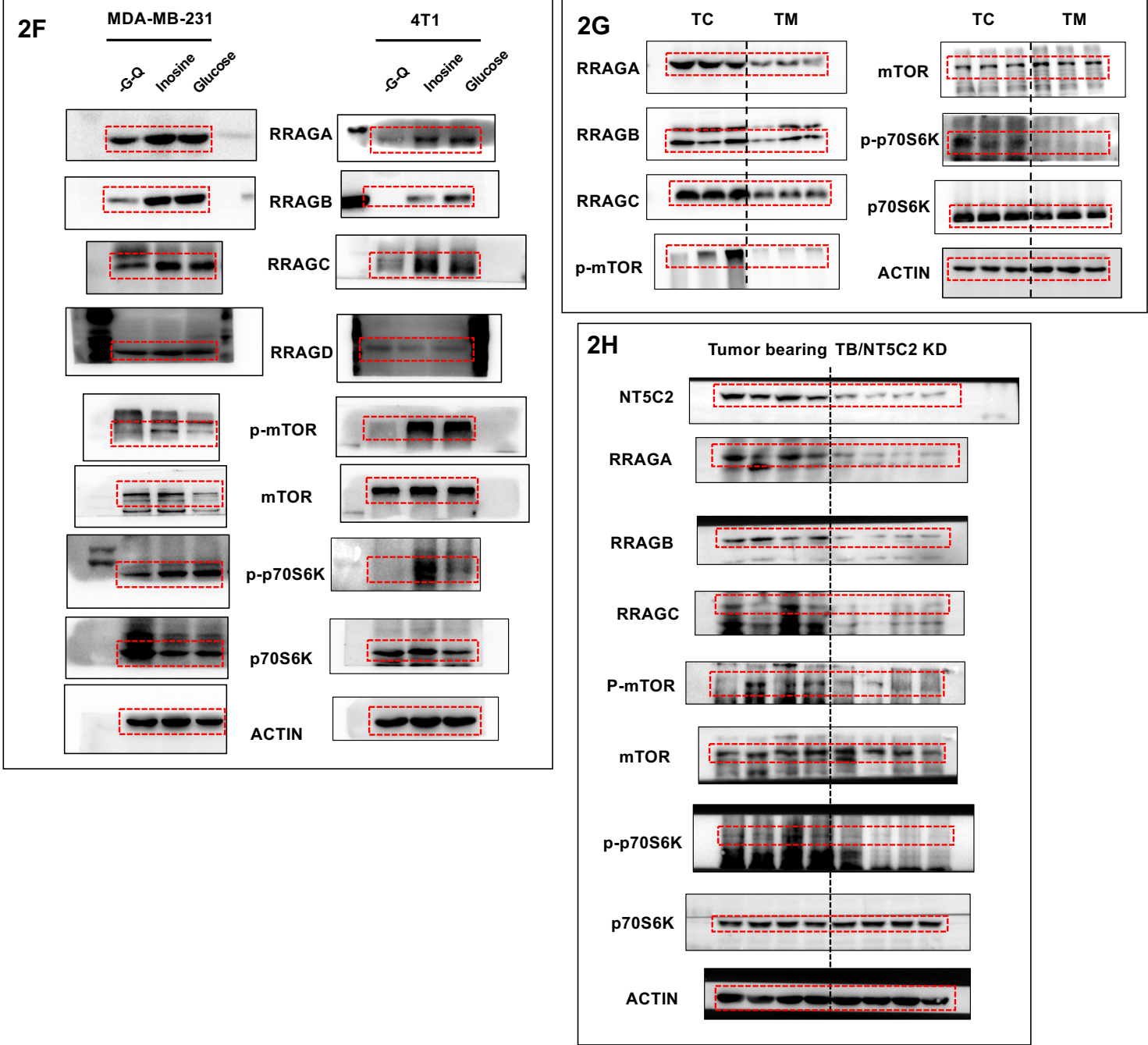

Figure S2

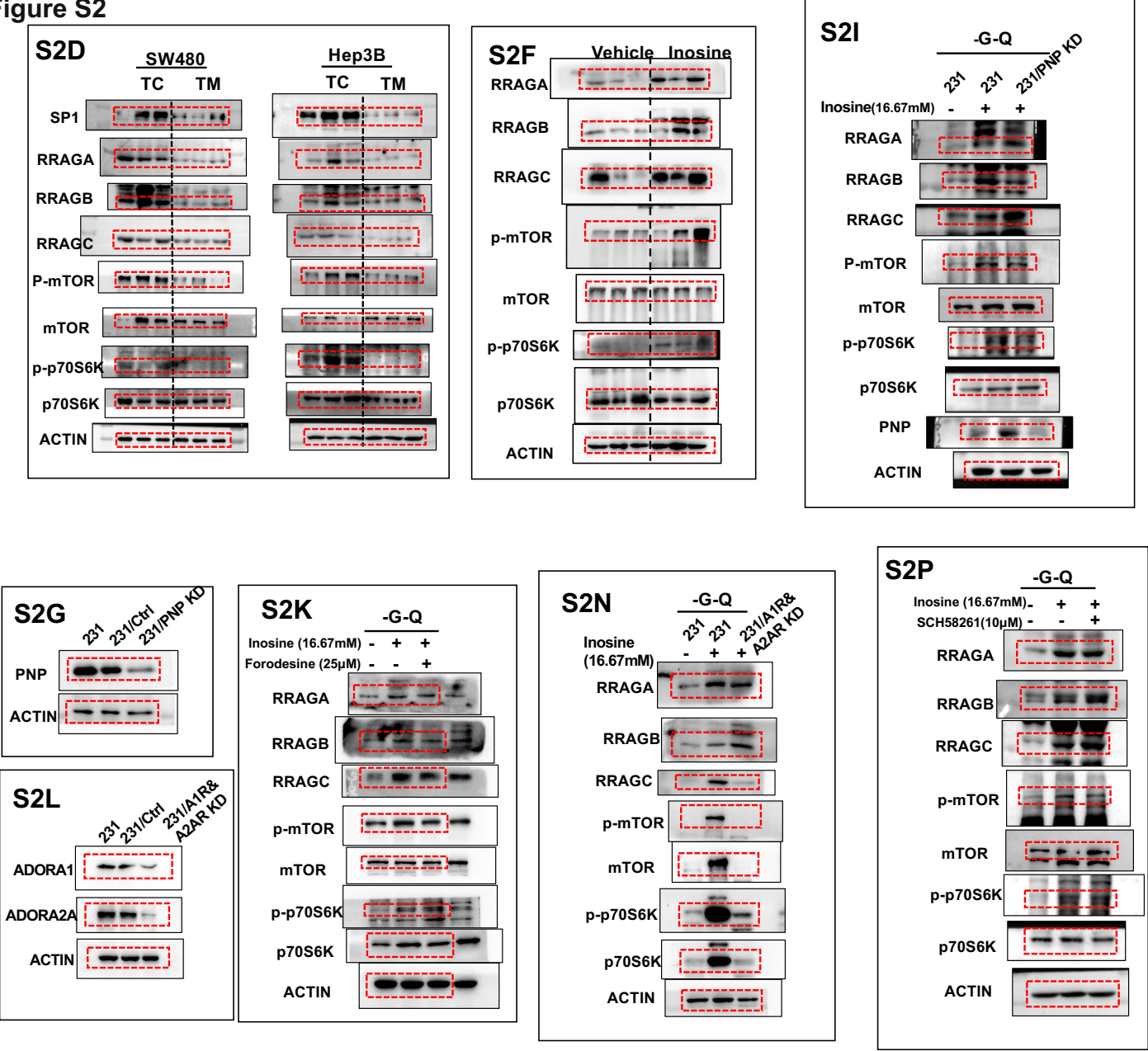

Figure 3

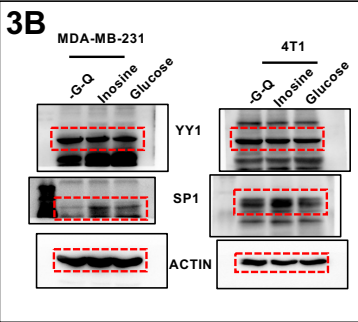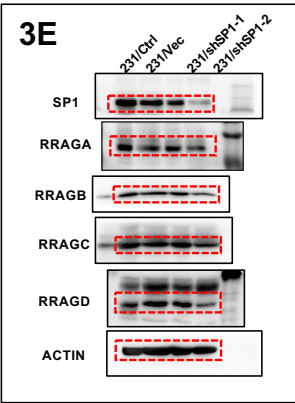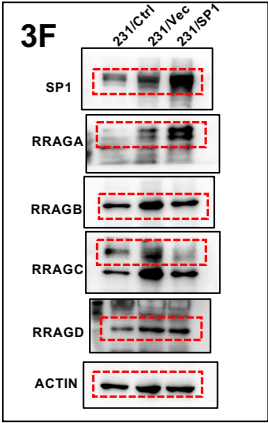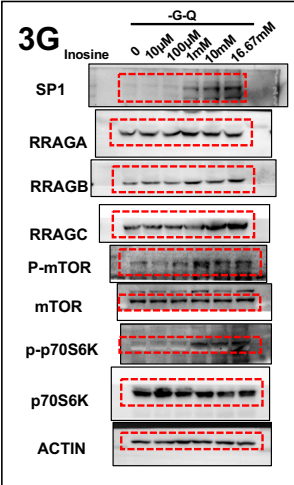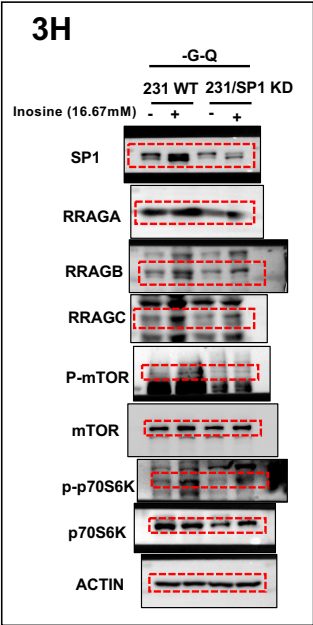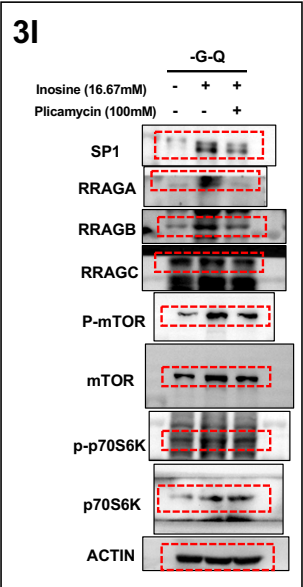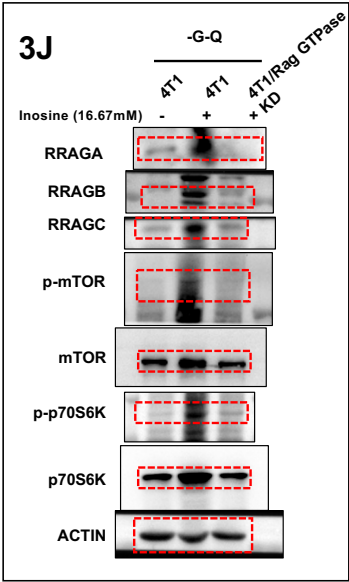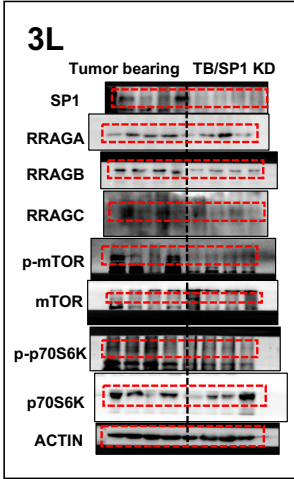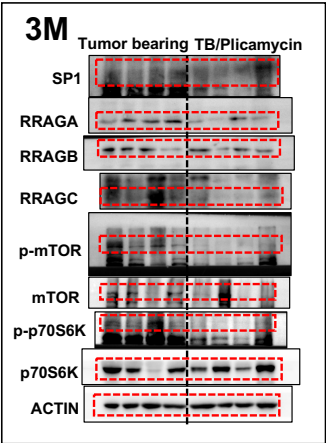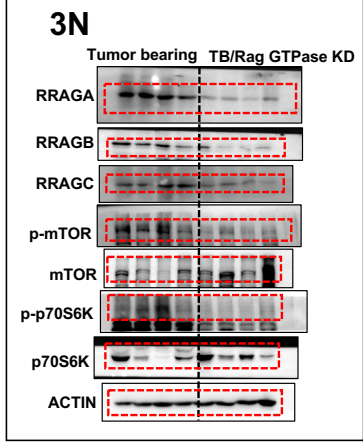

Figure S3

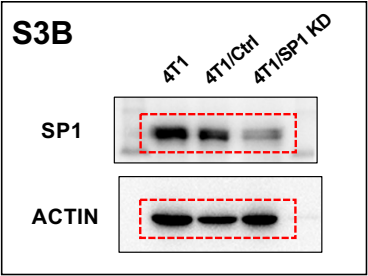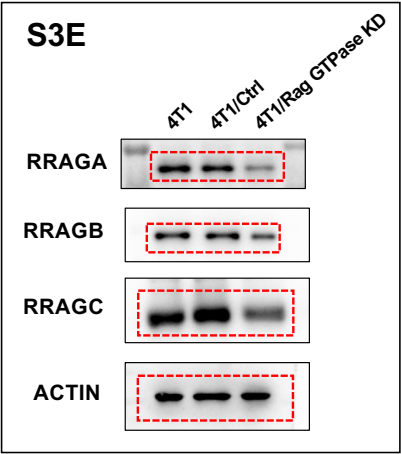

Figure 4

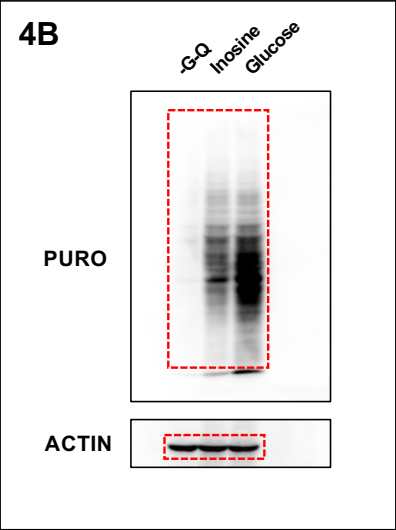

Figure S4

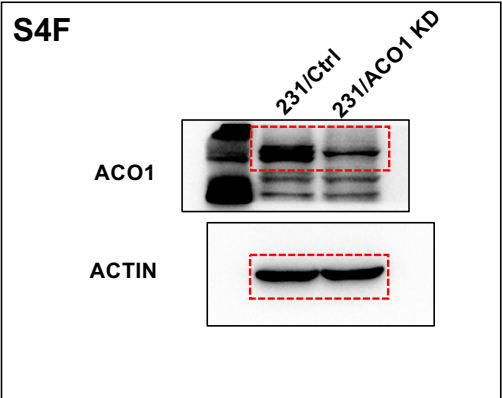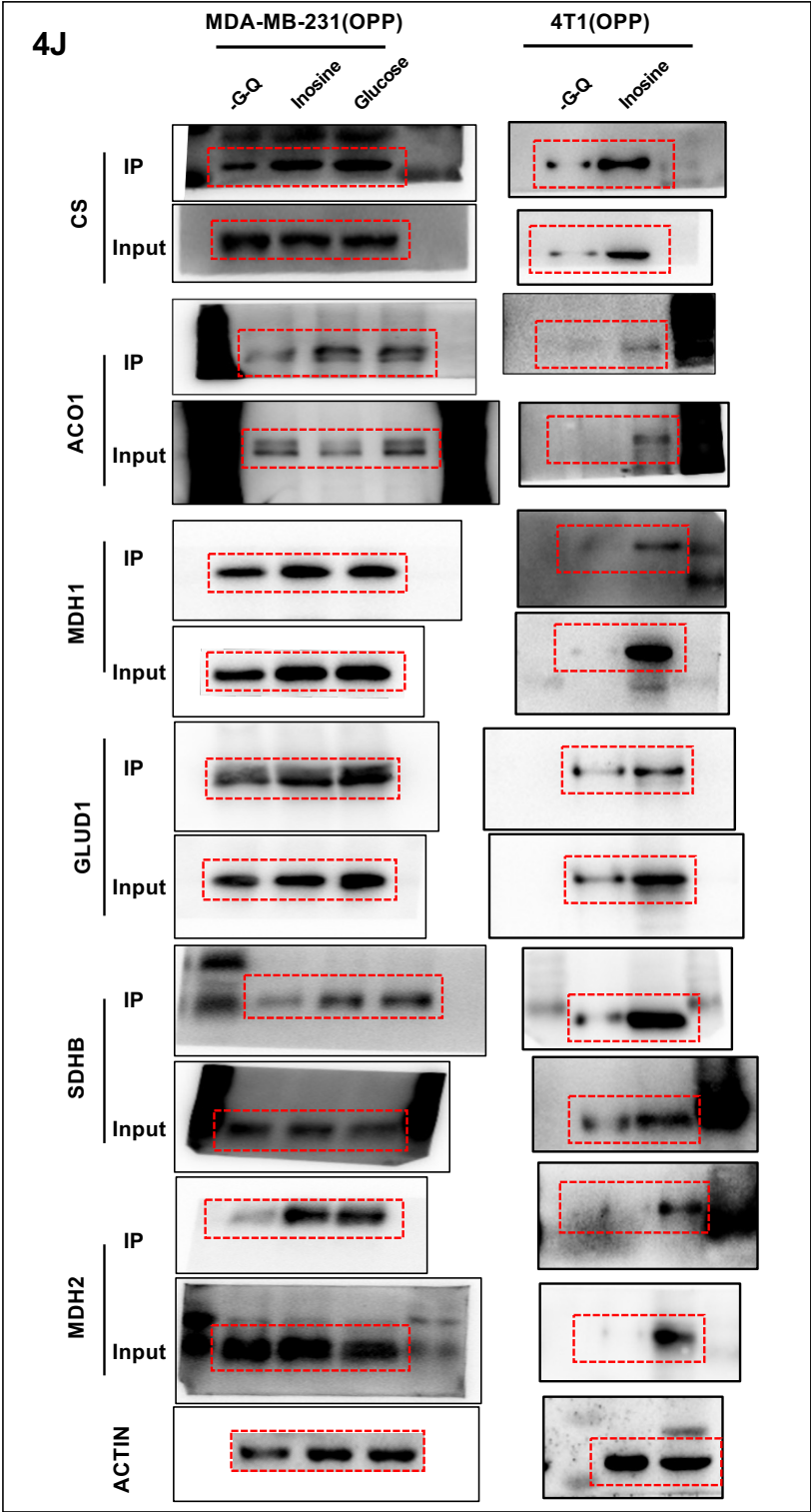

Figure 5

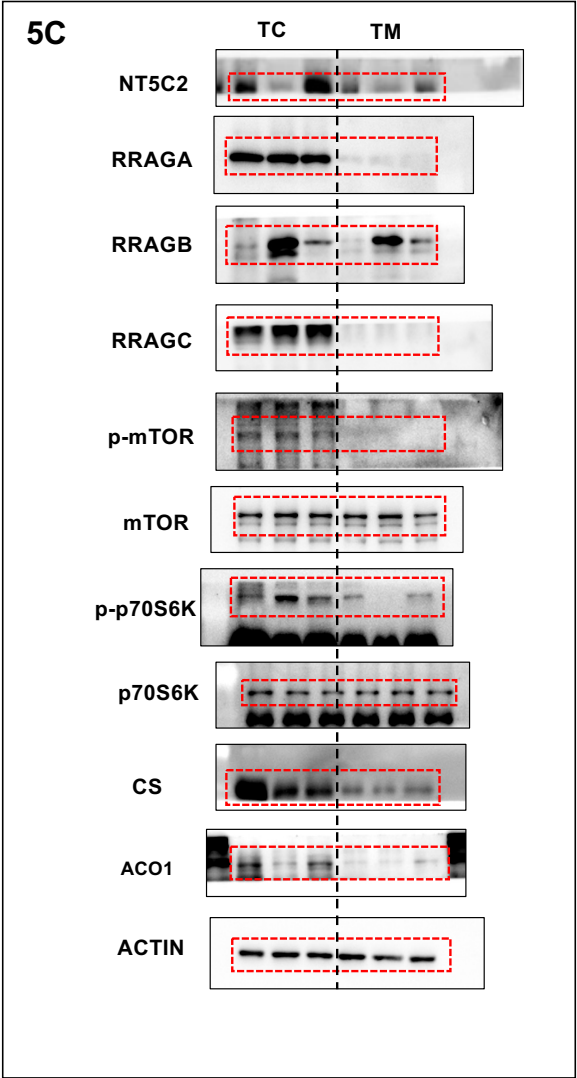

Supplement: Supplementary file 1 — Original Data File [file 41419_2023_6017_MOESM1_ESM.pdf]
