# Supplementary material for: Inosine enhances tumor mitochondrial respiration by inducing Rag GTPases and nascent protein synthesis under nutrient starvation
Source: Cell Death Dis. 2023 Aug 2;14(8):492. doi: 10.1038/s41419-023-06017-2 (PMC10397262; doi:10.1038/s41419-023-06017-2)
Supplement: Supplementary file 2 — Supplementary figures and tables [file 41419_2023_6017_MOESM2_ESM.docx]

**
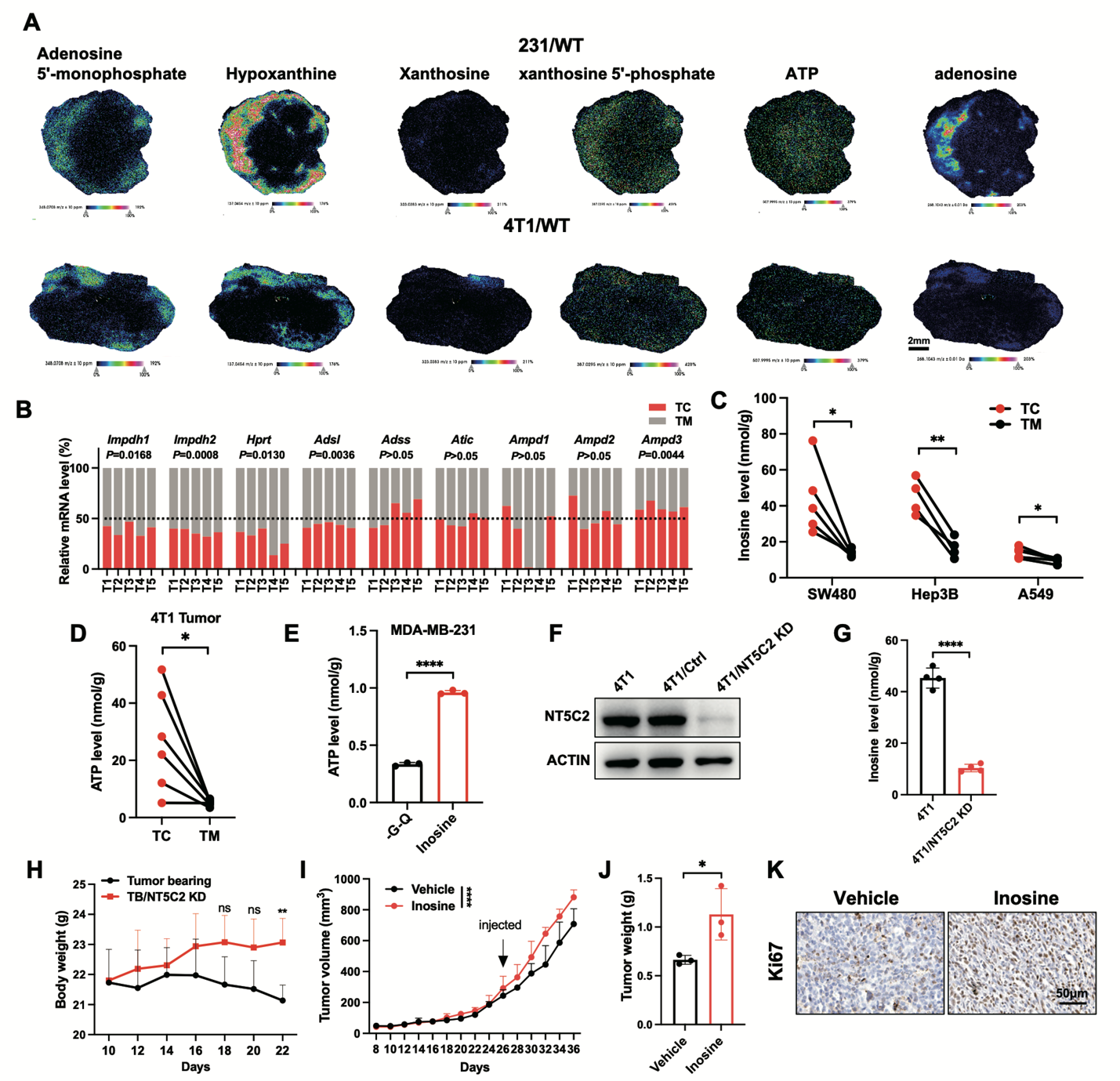
**

**Fig. S1 Elevation of inosine level promotes BC survival under glucose starvation.**

**(A)** Representative mass spectrometry imaging for indicated abundance in MDA-MB-231 and 4T1 wild type tumors from NSG mice and Balb/C mice. Scale bar, 2mm. **(B)** Quantification analysis of RNA level of 4T1 xenografted tumors in core and margin regions by RT-qPCR (paired two-tailed Student’s t-test, n = 5 mice per group). **(C)** Tissues from core regions of SW480, Hep3B and A549 cells xenografted tumors were used for inosine detection. Inosine concentrations were measured by ELISA kit (paired two-tailed Student’s t-test, n=5 mice for SW480 and A549, n=4 mice for Hep3B). **(D)** Tissues from core and margin regions of 4T1 cells xenografted tumors were used for ATP detection. ATP concentrations were measured by ELISA kit (paired two-tailed Student’s t-test, n = 6 mice per group). **(E)** MDA-MB-231 cells treated with glucose-glutamine-deficient medium (-G-Q) or supplemented with inosine were used for ATP detection. ATP concentrations were measured by ELISA kit (unpaired two-tailed Student’s t-test, n = 3 biological replicates). **(F)** Western blots showing the proteins levels in 4T1 cells with NT5C2 knockdown and relative control groups cells. **(G)** Detection of inosine levels in 4T1 and 4T1/NT5C2 KD cells. Inosine concentrations were measured by ELISA kit (unpaired two-tailed Student’s t-test, n = 4 biological replicates). **(H)** Body weight measurements of 4T1 cells and 4T1/NT5C2 KD cells xenografted tumors in Balb/C mice (unpaired two-tailed Student’s t-test, n=4 mice per group). **(I)** Tumors volume followed in indicated xenografted mice (two-way ANOVA, n=4 mice per group). **(J)** Tumor weight was monitored at the end of the experiment in indicated xenografted mice (unpaired two-tailed Student’s t-test, n=3 mice per group). **(K)** Representative IHC images showing Ki67 staining of core regions tissues were separated from indicated tumors. Scale bar, 50 μm.

**
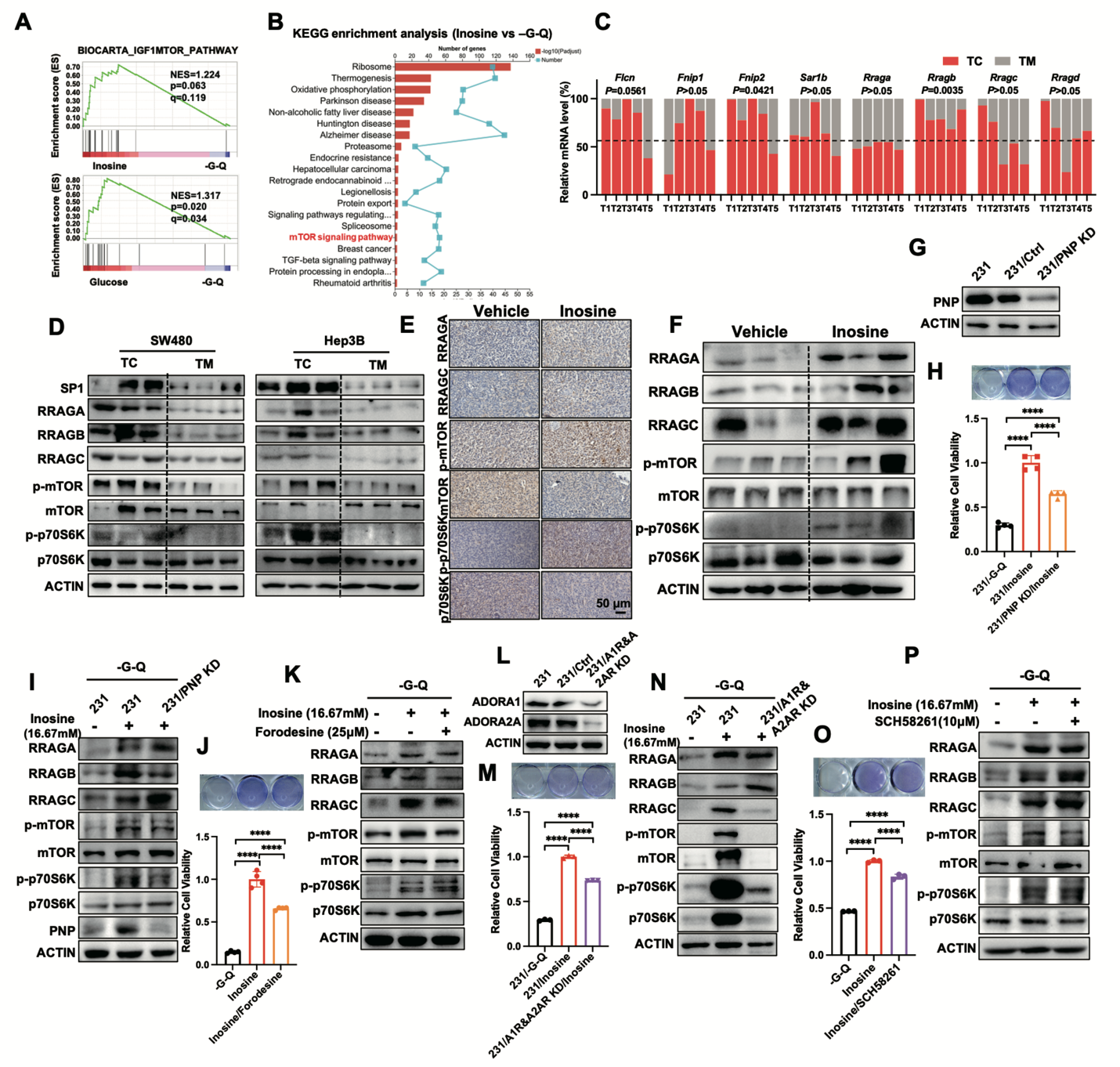
**

**Fig. S2 Inosine activates mTORC1 by inducing Rag GTPases.**

**(A)** MDA-MB-231 cells with indicated treatment were subjected to RNA-seq and GSEA, showing enrichment of genes related to indicated pathways (n=3 biological replicates per group). **(B)** The significantly enriched pathways (P<0.05) in the KEGG pathway analysis of the selected mRNAs according to inosine vs -G-Q treatment. **(C)** Quantification analysis of RNA levels in tissues from the core and margin regions were separated from 4T1 tumors by RT-qPCR (paired two-tailed Student’s t-test, n=5 mice per group). **(D)** Western blots showing the protein expression levels in the core and margin regions, tissues were separated from SW480 tumors and Hep3B tumors respectively. **(E)** Representative IHC images showing indicated protein staining of core regions, tissues were separated from 4T1 tumors injected with inosine and NaCl for control. Scale bar, 50 μm. **(F)** Western blots showing the proteins expression levels in the core regions, tissues were separated from 4T1 tumors injected with inosine and NaCl for control. **(G)** Western blots showing PNP protein level in MDA-MB-231 with PNP knockdown cells and relative control groups. **(H)** CCK8 assay and crystal violet staining results showing the cell viability of MDA-MB-231 and MDA-MB-231/PNP KD cells treated with glucose-glutamine-deficient medium (-G-Q) or supplemented with inosine (one-way ANOVA, n = 4 biological replicates). **(I)** Western blots showing the proteins expression levels in MDA-MB-231 and MDA-MB-231/PNP KD cells treated with glucose-glutamine-deficient medium (-G-Q) or supplemented with inosine. **(J)** CCK8 assay and crystal violet staining results showing the cell viability of MDA-MB-231 cells with Forodesine treatment in indicated medium (one-way ANOVA, n = 4 biological replicates). **(K)** Western blots showing the proteins expression levels in MDA-MB-231 cells with Forodesine treatment in indicated medium**. (L)** Western blots showing ADORA1 and ADORA2A protein levels in MDA-MB-231 with ADORA1 and ADORA2A knockdown cells and relative control groups. **(M)** CCK8 assay and crystal violet staining results showing the cell viability of MDA-MB-231 and MDA-MB-231/ADORA1&ADORA2A knockdown cells treated with glucose-glutamine-deficient medium (-G-Q) or supplemented with inosine (one-way ANOVA, n = 3 biological replicates). **(N)** Western blots showing the proteins expression levels in MDA-MB-231 and MDA-MB-231/ADORA1&ADORA2A knockdown cells treated with glucose-glutamine-deficient medium (-G-Q) or supplemented with inosine. **(O)** CCK8 assay and crystal violet staining results showing the cell viability of MDA-MB-231 cells with SCH58261 treatment in indicated medium (one-way ANOVA, n = 3 biological replicates). **(P)** Western blots showing the proteins expression levels in MDA-MB-231 cells with SCH58261 treatment in indicated medium.

**
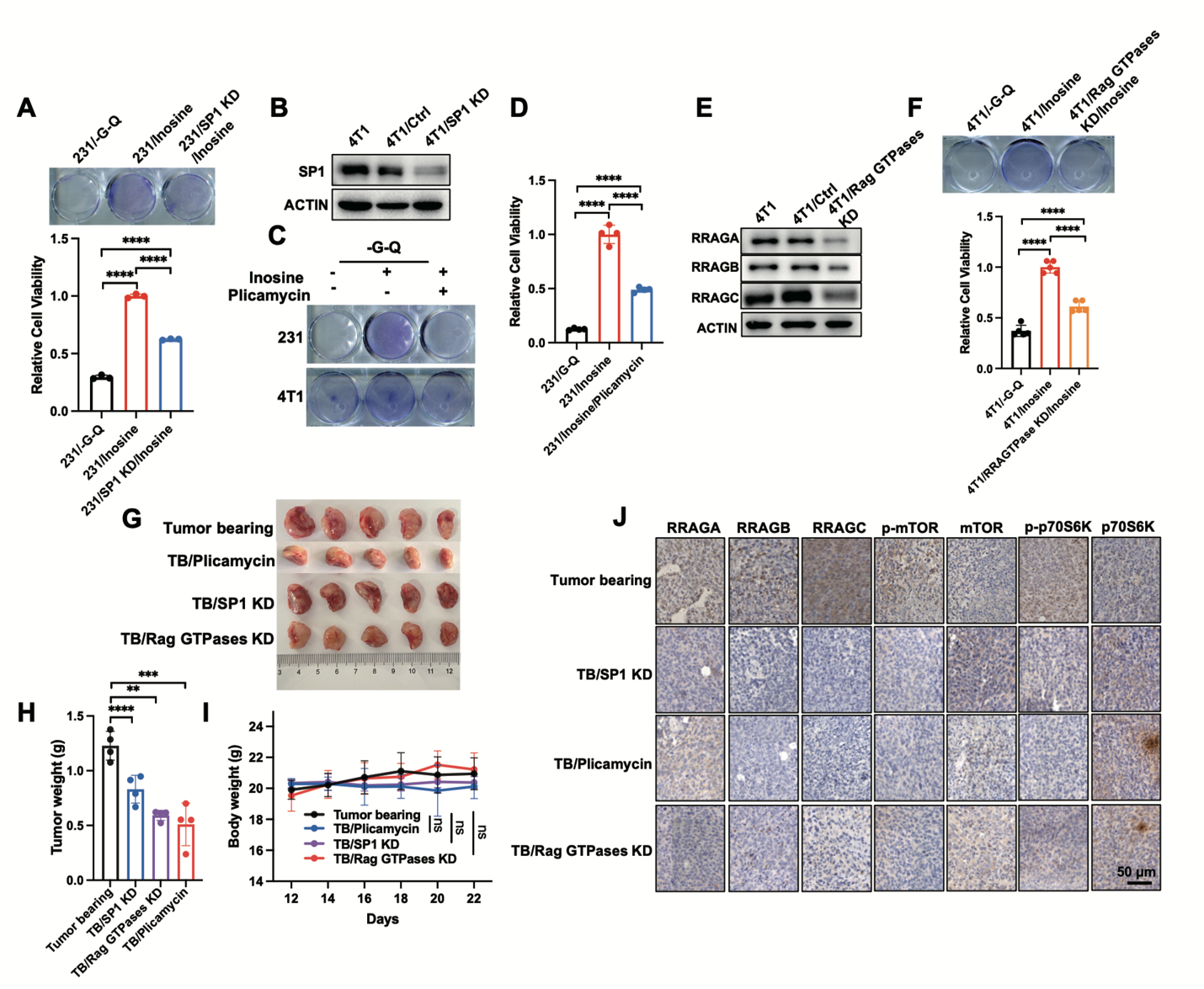
**

**Fig. S3 Inosine induces Rag GTPases expression by elevating the level of SP1.**

**(A)** The crystal violet staining results showing proliferation of MDA-MB-231/SP1 KD cells treated with glucose-glutamine-deficient medium (-G-Q) or supplemented with inosine. CCK8 assay results showing the effect of knockdown of SP1 on cell growth in MDA-MB-231 cells treated with glucose-glutamine-deficient medium (-G-Q) or supplemented with inosine (one-way ANOVA, n = 3 biological replicates). **(B)** Western blots showing the proteins levels in 4T1 cells with SP1 knockdown and relative control groups cells. **(C)** The crystal violet staining results showing proliferation of MDA-MB-231 and 4T1 cells treated with glucose-glutamine-deficient medium (-G-Q) or supplemented with inosine and plicamycin. **(D)** CCK8 assay results showing the effect of plicamycin (SP1 inhibitor) on cell growth in MDA-MB-231 cells treated with glucose-glutamine-deficient medium (-G-Q) or supplemented with inosine (one-way ANOVA, n = 4 biological replicates). **(E)** Western blots showing the proteins levels in 4T1 cells with Rag GTPases knockdown and relative control groups cells. **(F)** The crystal violet staining results and CCK8 assay results showing proliferation of 4T1 cells and 4T1/Rag GTPases KD cells treated with glucose-glutamine-deficient medium (-G-Q) or supplemented with inosine (one-way ANOVA, n = 5 biological replicates). **(G)** Representative images of indicated xenografted tumors in Balb/C mice were shown (n=5 mice per group). **(H)** Tumor weight of indicated xenografted tumors in Balb/C mice in indicated groups (one-way ANOVA, n = 4 mice per group). **(I)** Body weight measurements of 4T1 xenografted in Balb/C mice in indicated groups (two-way ANOVA, n=4 mice per group). **(J)** Representative IHC images showing indicated proteins staining of core regions tissues were separated from 4T1 tumors, 4T1/SP1 KD tumors, 4T1/plicamycin tumors and 4T1/Rag GTPase tumors. Scale bar, 50 μm.

**
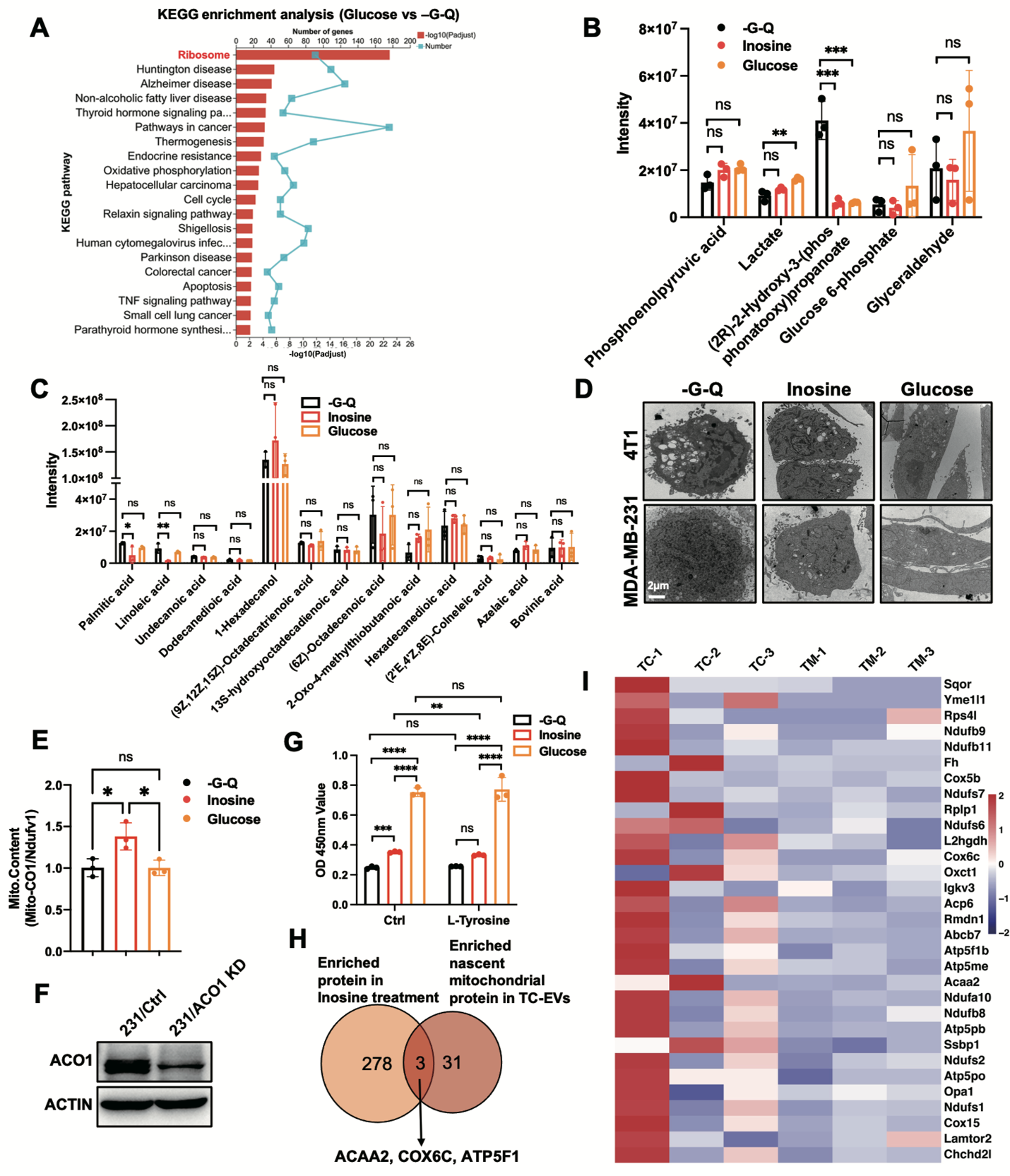
**

**Fig. S4 Inosine induces nascent protein synthesis to enhance mitochondrial respiration.**

**(A)** The significantly enriched pathways (P<0.05) in the KEGG pathway analysis of the selected mRNAs according to glucose vs -G-Q treatment. **(B)** Abundance of metabolites during glycolysis in MDA-MB-231 cells with -G-Q, inosine and glucose treatment (one-way ANOVA, n = 3 biological replicates). **(C)** Abundance of metabolites during fatty acid metabolism in MDA-MB-231 cells with -G-Q, inosine and glucose treatment (one-way ANOVA, n=3 biological replicates). **(D)** Electron microscopy analysis of MDA-MB-231 and 4T1 cells with indicated treatment. Scale bar, 2 μm. **(E)** Mitochondria contents in MDA-MB-231 cells under different treatment (one-way ANOVA, n = 3 biological replicates). **(F)** Western blots showing the effect of ACO1 knockdown in MDA-MB-231 cells. **(G)** CCK8 assay results showing the effect of CS inhibitor (4 mM L-Tyrosine) on cell growth in MDA-MB-231 cells treated with inosine when compared to control (one-way ANOVA and unpaired two-tailed Student’s t-test, n = 3 biological replicates). **(H)** Venn diagram shows overlapping nascent proteins enriched in both inosine treatment and mitochondrial protein in EVs derived from TC compared with TM. **(I)** The level of proteins in EVs from core and margin of tumors in mitochondria shown as a heat map.

**
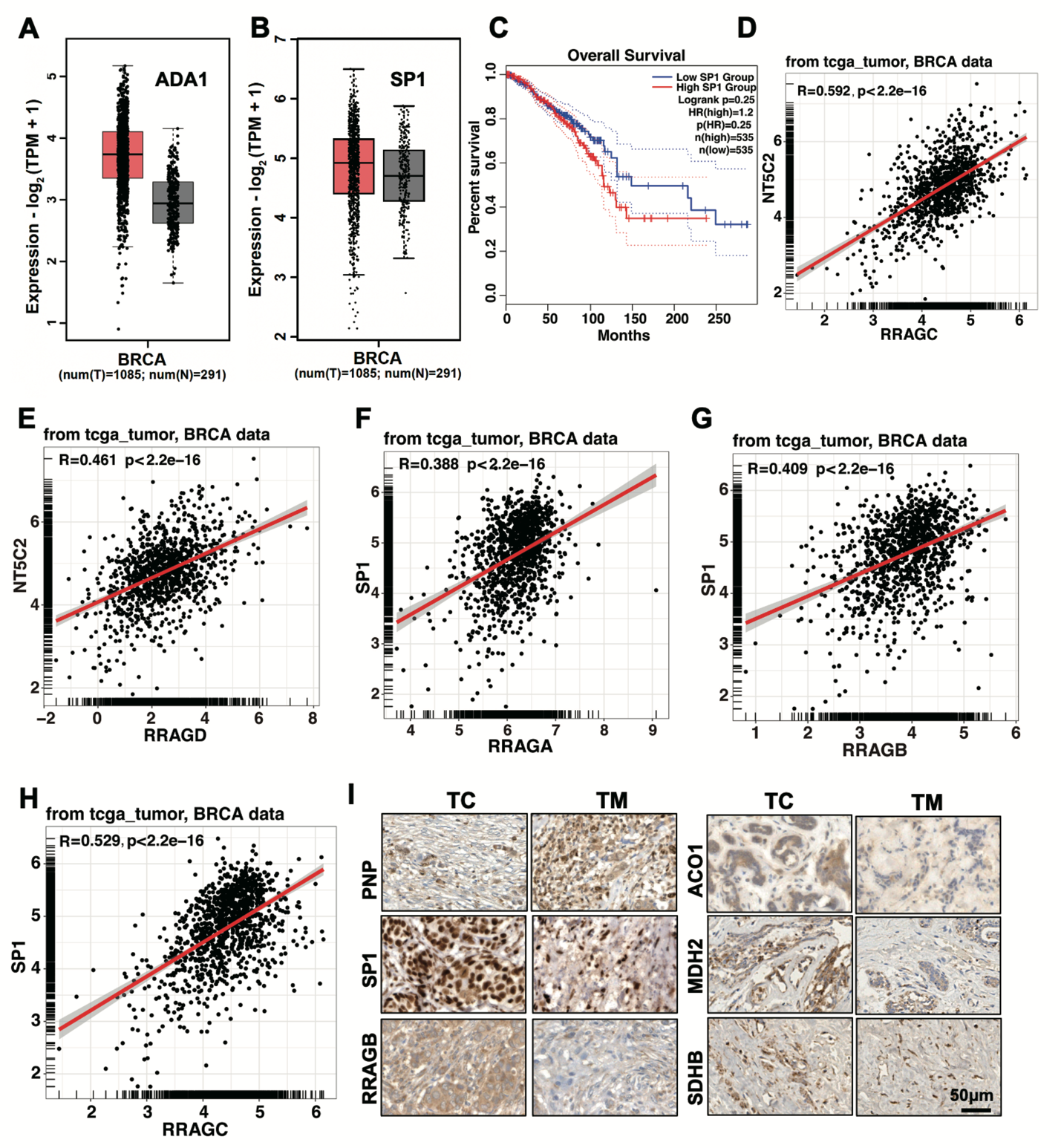
**

**Fig. S5 Inosine mediated Rag GTPases expression widely exists in human breast tumors.**

**(A-B)** Expression levels of ADA1 and SP1 in BRCA tumors samples from the TCGA database (n=1085 for tumor, n=291 for normal). **(C)** Kaplan-Meier survival curves showing relationship between high and low levels in SP1 expression and overall survival in patients with breast cancer. **(D-E)** Correlations between NT5C2 expression and the expression of RRAGC and RRAGD in patients with breast cancer. **(F-H)** Correlations between RRAGA, RRAGB, RRAGC and SP1 expression in patients with breast cancer. **(I)** Representative IHC images showing indicated proteins staining of core and margin regions tissues were separated from BRCA tumors. Scale bar, 50 μm.

**Supplementary Table 1. Primer sequences used in this study.**

| **Gene** | **Forward primer** | **Reverse primer** |
| --- | --- | --- |
| **Human *RRAGA*** | ACACTCCCACGTCCGATTC | CTCTCCACGTCAAACACGTAA |
| **Human *RRAGB*** | AACCGGAAGGATCGCTTGG | CGACGTGTGTCTCTGGCAA |
| **Human *RRAGC*** | TCCACTGTTTCCGAAAAGCTATT | CGTGCCATTGTGTGTCAGC |
| **Human *RRAGD*** | CTAGCGGACTACGGAGACG | ATGAGCAGGATTCTCGGCTTC |
| **Human *ACTIN*** | ACAGAGCCTCGCCTTTGC | CCACCATCACGCCCTGG |
| **Human *ADA1*** | GCCTTCGACAAGCCCAAAGTA | CTCTGCTGTGTTAGCTGGGAG |
| **Human *SAR1B*** | TTGGATTTACAGTGGTTTCAGC | GTCAGTTCTTCGGAAGTGGGA |
| **Human *FLCN*** | TCTTCAGCATTGTCCGCCAG | AGTTGATGAGGTAGATCCGGTC |
| **Human *FNIP1*** | GGTTCTCGGTGCTCTTCTGAT | GCTGTGGAGGGGAACGAAT |
| **Human *FNIP2*** | TCTTCTCTGCTAGAATGGGCA | AGTACGACAAGGACCCAAACTA |
| **Human *SP1*** | GTGGAGGCAACATCATTGCTG | GCCACTGGTACATTGGTCACAT |
| **Human *YY1*** | CCTCTCAGATCCCAAACAACTG | GCCTTTATGAGGGCAAGCTATT |
| **Human *PNP*** | TTTCCCCGAAGTACAGTGCC | CCCTCACTGGGAATGTCACC |
| **Human *NT5C2*** | GATTGTTGCGGTGCGCTG | CGGTTCACAAACACCCGATG |
| **Human *E2F6*** | TCCATGAACAGATCGTCATTGC | TCCGTTGGTGCTCCTTATGTG |
| **Human *USF2*** | CTGTGATCCAAAATCCCTTCAGC | GGTCTGTGGTCTGTACGGAC |
| **mouse *Sp1*** | AGGGTCCGAGTCAGTCAGG | CTCGCTGCCATTGGTACTGTT |
| **mouse *Yy1*** | GTGGTTGAAGAGCAGATCATTGG | TTGCTTAGGGTCTGAGAGGTC |
| **mouse *Rraga*** | CGCGAACTGGAAAAAGATATGC | AAGATTTTGGCGTCAGGTGAA |
| **mouse *Rragc*** | CCCAAGGACTTCGGCTACG | GGAGCCCCATAAGCAGGAT |
| **mouse *Actin*** | CGAGGCCCAGAGCAAGAGAG | CGGTTGGCCTTAGGGTTCAG |
| **mouse *Nt5c2*** | ACGACCTCCTGGAGTGACC | GGAGACTTGTACACGGCTAGG |
| **mouse *Ada1*** | AGAGGATCGCCTACGAGTTTG | TTGGGTCCACCTTGGAATTGG |
| **mouse *Pnp*** | GCAGATGCTGTTGGCATGAG | AGCACTCCATTGCAGGTCAG |
| **mouse *Sar1b*** | TGCCGGGAAAACAACTTTGC | TCCATACTCTTCGGGCTTGC |
| **mouse *Impdh1*** | GGCTACGTTCCCGAGGATG | GGCTGATGTCAGGTCCACTT |
| **mouse *Impdh2*** | GCGCTTACAGGCGGTATTG | AAAACATCCCGCACGCGAT |
| **mouse *Hprt*** | TCAGTCAACGGGGGACATAAA | GGGGCTGTACTGCTTAACCAG |
| **mouse *Adsl*** | AGCCGCGAGATGTGTTTCTT | TCAATGTTGTTCAGGTTCGACTT |
| **mouse *Adss*** | ACACGGGGTAGAGAATTTGGA | GGTAAGGGCCAACGCAGTA |
| **mouse *Atic*** | GCCTCGTGGAATTTGCCAGA | AACCCTGTTAGCTCAGACACA |
| **mouse *Ampd1*** | CATCCCCAACCTACGAGAGTG | TGCCCGATAGAGACCTTTACAAA |
| **mouse *Ampd2*** | CCTCTCCGCTACAGTCTGC | CTCCTTGCATTTGCCATCCAT |
| **mouse *Ampd3*** | GTTGGCGGAGAAGGTGTTTG | CTGCGACCGGATCATCTTGAA |
| **mouse *Flcn*** | CTTACCGGCTTCAAGTCTCTTC | TTCAAACGCTGAATGGACCAG |
| **mouse *Fnip1*** | ATCGAAGCTCTGTAATGATGCTC | AACCCTGGTATTTGGGACACT |
| **mouse *Fnip2*** | TACAACACGCTAAGCAACAGC | TTGTGGAGGAGATCGTATGTAGT |
| **mouse *Sar1b*** | TGCCGGGAAAACAACTTTGC | TCCATACTCTTCGGGCTTGC |
| **mouse *Rragb*** | CTAGCCAGCGTGACAACATCT | GCCTCAAGGCATGACTGATAGT |
| **mouse *Rragd*** | AGGAGCGGCAAGTCGTCTAT | CCGGCAGATCCTGTTGGTG |

**Supplementary Table 2. Metabolites identified by mass spectrometry imaging**.

| **Formula** | **CAS** | **Name** | **Maximum mean value - 231WT - Root Mean Square** | **Maximum mean value - 4T1WT - Root Mean Square** | **Maximum mean value - TWBC - Root Mean Square** |
| --- | --- | --- | --- | --- | --- |
| C5H4N4O | 68-94-0 | Hypoxanthine | 15.36911678 | 3.980580091 | 3.507127047 |
| C10H12N4O6 | 146-80-5 | Xanthosine | 0.634128153 | 0.791768253 | 0.325643957 |
| C10H14N5O7P | 61-19-8 | Adenosine 5'-monophosphate | 1.618283749 | 1.580064654 | 3.779464006 |
| C10H13N4O9P | 523-98-8 | xanthosine 5'-phosphate | 0.692597747 | 0.406159788 | 0.45317784 |
| C10H16N5O13P3 | 56-65-5 | ATP | 0.449055582 | 0.184164733 | 0.759664476 |
| C10H13N4O8P | 131-99-7 | Inosine 5'-monophosphate | 4.776593208 | 3.441194057 | 1.450395346 |
| C10H13N5O4 | 58-61-7 | Adenosine | 10.27423286 | 2.237029791 | 6.224448204 |

| **Supplementary Table 3. OPP** **labeled nascent proteins identified by LC-MS/MS.** | | | | | | | | | |  |  |  |
| --- | --- | --- | --- | --- | --- | --- | --- | --- | --- | --- | --- | --- |
|  | **Glucose-1** | **Glucose-2** | **Glucose-3** | **Inosine-1** | **Inosine-2** | **Inosine-3** | **-G-Q-1** | **-G-Q-2** | **-G-Q-3** |  |  |  |
| **ALDOA** | 2.10E+09 | 6.90E+08 | 2.00E+08 | 1.00E+08 | 5.10E+08 | 4.10E+08 | 5.70E+07 | 7.90E+08 | 1.80E+08 |  |  |  |
| **CS** | 2.80E+07 | 1.90E+07 | 3.80E+07 | 1.90E+07 | 2.30E+07 | 1.50E+07 | 1.10E+07 | 1.70E+07 | 3.00E+07 |  |  |  |
| **ECHS1** | 2.60E+07 | 8.00E+07 | 6.50E+07 | 5.10E+07 | 7.80E+07 | 4.30E+07 | 1.60E+07 | 2.00E+07 | 2.70E+07 |  |  |  |
| **ENO1** | 2.90E+08 | 2.00E+08 | 2.70E+08 | 1.50E+08 | 2.30E+08 | 1.50E+08 | 4.40E+07 | 1.10E+08 | 1.30E+08 |  |  |  |
| **GAPDH** | 7.00E+09 | 5.60E+09 | 2.70E+09 | 5.60E+08 | 6.70E+09 | 5.30E+09 | 1.00E+09 | 3.30E+09 | 2.60E+09 |  |  |  |
| **GLUD1** | 1.90E+07 | 2.00E+07 | 2.00E+07 | 6.80E+06 | 2.00E+07 | 1.60E+07 | 8.40E+06 | 1.10E+07 | 1.30E+07 |  |  |  |
| **GOT2** | 1.20E+08 | 2.60E+07 | 3.50E+07 | 2.00E+07 | 3.90E+07 | 2.20E+07 | 1.00E+07 | 2.90E+07 | 1.80E+07 |  |  |  |
| **GPI** | 3.10E+07 | 3.10E+07 | 3.50E+07 | 1.30E+07 | 2.10E+07 | 2.00E+07 | 9.60E+06 | 1.20E+07 | 1.20E+07 |  |  |  |
| **MDH1** | 6.70E+08 | 4.50E+08 | 1.30E+08 | 2.50E+07 | 4.80E+08 | 4.10E+08 | 4.60E+07 | 2.80E+08 | 1.70E+08 |  |  |  |
| **MDH2** | 8.70E+08 | 7.90E+08 | 4.90E+08 | 6.10E+07 | 9.20E+08 | 7.60E+08 | 2.40E+08 | 5.50E+08 | 4.70E+08 |  |  |  |
| **PGAM1** | 5.30E+08 | 1.50E+09 | 1.60E+09 | 8.50E+08 | 1.40E+09 | 6.70E+08 | 3.20E+08 | 4.40E+08 | 4.90E+08 |  |  |  |
| **PGK1** | 7.30E+07 | 5.20E+07 | 8.20E+07 | 2.80E+07 | 6.50E+07 | 5.30E+07 | 1.60E+07 | 2.70E+07 | 4.10E+06 |  |  |  |
| **PKM** | 4.70E+08 | 3.90E+08 | 3.70E+08 | 2.80E+08 | 3.50E+08 | 2.90E+08 | 1.30E+08 | 1.80E+08 | 2.30E+08 |  |  |  |
| **PRPS1** | 5.80E+07 | 1.50E+08 | 4.90E+07 | 4.90E+06 | 8.50E+07 | 1.00E+08 | 3.30E+07 | 3.40E+07 | 4.70E+07 |  |  |  |
| **PRPS2** | 6.30E+07 | 6.20E+07 | 5.00E+07 | 5.20E+06 | 9.60E+07 | 7.80E+07 | 1.60E+07 | 4.00E+07 | 3.30E+07 |  |  |  |
| **SDHB** | 2.00E+07 | 3.60E+07 | 6.00E+07 | 1.80E+07 | 4.80E+07 | 1.70E+07 | 1.30E+07 | 1.10E+07 | 1.30E+07 |  |  |  |
| **SHMT2** | 1.20E+07 | 2.10E+07 | 1.50E+07 | 1.00E+07 | 1.30E+07 | 1.30E+07 | 5.70E+06 | 7.20E+06 | 7.40E+06 |  |  |  |
| **TALDO1** | 2.80E+08 | 1.20E+08 | 4.40E+07 | 1.30E+07 | 1.00E+08 | 9.20E+07 | 1.40E+07 | 1.10E+08 | 5.60E+07 |  |  |  |
| **TPI1** | 1.00E+09 | 1.60E+09 | 2.00E+09 | 1.50E+09 | 2.00E+09 | 1.30E+09 | 3.60E+08 | 4.10E+08 | 7.30E+08 |  |  |  |
| **SUCLG1** | 1.00E+08 | 1.10E+08 | 8.50E+07 | 9.80E+06 | 1.20E+08 | 9.20E+07 | 2.50E+07 | 5.90E+07 | 5.40E+07 |  |  |  |
| **PGLS** | 3.40E+07 | 4.60E+07 | 7.00E+07 | 3.40E+07 | 6.20E+07 | 2.60E+07 | 1.20E+07 | 1.70E+07 | 1.60E+07 |  |  |  |
| **MAT2B** | 9.20E+07 | 2.90E+07 | 1.80E+07 | 4.00E+06 | 3.40E+07 | 3.00E+07 | 3.20E+06 | 2.60E+07 | 5.40E+06 |  |  |  |
| **ALDH9A1** | 1.30E+07 | 1.00E+07 | 1.20E+07 | 9.00E+06 | 9.80E+06 | 6.40E+06 | 5.20E+06 | 5.20E+06 | 7.80E+06 |  |  |  |
| **LDHA** | 4.80E+09 | 4.20E+09 | 3.20E+09 | 2.70E+08 | 5.30E+09 | 4.60E+09 | 9.90E+08 | 1.90E+09 | 1.60E+09 |  |  |  |
| **LDHB** | 1.80E+09 | 1.40E+09 | 4.00E+08 | 9.30E+07 | 1.30E+09 | 1.30E+09 | 1.50E+08 | 8.80E+08 | 5.20E+08 |  |  |  |
| **RPS6** | 7.90E+08 | 8.60E+08 | 6.50E+08 | 1.00E+08 | 8.70E+08 | 8.00E+08 | 3.00E+08 | 2.50E+08 | 2.20E+08 |  |  |  |
| **ELOB** | 1.70E+07 | 1.40E+07 | 2.00E+07 | 2.50E+07 | 6.60E+06 | 1.40E+07 | 1.40E+07 | 1.70E+07 | 1.60E+07 |  |  |  |
| **TFRC** | 2.20E+07 | 1.40E+07 | 2.40E+07 | 1.30E+07 | 1.10E+07 | 1.10E+07 | 9.90E+06 | 2.90E+06 | 7.20E+06 |  |  |  |

**Supplementary Table 4. Determination of metabolic composition by LC-MS/MS.**

| **Name** | **-G-Q-1** | **-G-Q-2** | **-G-Q-3** | **Inosine-1** | **Inosine-2** | **Inosine-3** | **Glucose-1** | **Glucose-2** | **Glucose-3** |
| --- | --- | --- | --- | --- | --- | --- | --- | --- | --- |
| **Succinic acid** | 16108777.19 | 17048264.57 | 10083099.28 | 8824163.697 | 2640574.472 | 2594347.86 | 2151657.858 | 11748943.9 | 9139442.391 |
| **Fumaric acid** | 24933987.58 | 4947763.499 | 20615672.65 | 3712695.589 | 16105848.92 | 4773444.59 | 14359064.7 | 24573182.2 | 4250580.507 |
| **Isocitric acid** | 2647110.225 | 2400793.37 | 1697665.939 | 6572198.062 | 8501453.109 | 8239584.28 | 7850533.743 | 10894079.2 | 9766185.004 |
| **Oxalosuccinic acid** | 4142638.867 | 5259194.216 | 4120583.03 | 3899821.373 | 4590913.328 | 4987810.48 | 4346495.77 | 4977920.04 | 5769504.134 |
| **L-Malic acid** | 28297982.67 | 28019645.31 | 25820362.31 | 41907106.38 | 18553097.62 | 17318803.1 | 36575952.35 | 15319502.1 | 14518769.31 |
| **Pyrophosphate** | 86948523.01 | 114511719.3 | 89444529.64 | 136796407.1 | 203937532.7 | 174268347 | 159059247 | 175364624 | 165873737.2 |
| **Phosphoenolpyruvic acid** | 13547538.63 | 18187550.69 | 12275447.23 | 16979193.44 | 20579904.89 | 22612225.5 | 22735391.09 | 20049170.2 | 19808763.38 |
| **Lactate** | 10884817.37 | 6653828.677 | 9994714.078 | 12778243.18 | 11353925 | 11620269.1 | 16226275.42 | 15438501.7 | 16962101.22 |
| **(2R)-2-Hydroxy-3-(phosphonatooxy)propanoate** | 34947053.93 | 50318698.03 | 38040319.77 | 5741394.743 | 4940895.964 | 8022861.19 | 5900705.489 | 6457737.74 | 6326256.895 |
| **Glucose 6-phosphate** | 2108692.391 | 7495133.073 | 6768769.689 | 4125705.903 | 7029331.065 | 1008428.61 | 6047896.78 | 28636432.2 | 5595312.711 |
| **Glyceraldehyde** | 7340461.333 | 33128212.77 | 21974116.29 | 5865500.16 | 21047410.97 | 20794961.7 | 54512868.46 | 48124932.2 | 7301569.705 |
| **Palmitic acid** | 12898613.9 | 11872629.59 | 12927365.03 | 2001011.112 | 2178068.092 | 10903404.7 | 10530330.65 | 9771146.79 | 8802934.703 |
| **Linoleic acid** | 6825414.222 | 8312462.374 | 12448161.06 | 1108865.605 | 1729619.443 | 1384585.68 | 7591630.455 | 6855653.65 | 6308375.086 |
| **Undecanoic acid** | 4125711.485 | 4049530.323 | 4367472.866 | 3765517.383 | 3923152.079 | 3612848.55 | 4144541.132 | 3622159.52 | 3304227.038 |
| **Dodecanoic acid** | 69915072.31 | 37759765.07 | 41807302.19 | 103177446.2 | 396277281 | 81596244.1 | 12766457.09 | 25122925.7 | 14847855.21 |
| **1-Hexadecanol** | 121087590.4 | 132530604.1 | 151018133.1 | 240919401.8 | 177822994.1 | 97749909.7 | 133309539.7 | 142754318 | 104599772.1 |
| **(9Z,12Z,15Z)-Octadecatrienoic acid** | 13249896.12 | 12004137.3 | 12873262.92 | 11401351.87 | 11598888.22 | 10636209.3 | 10568822.8 | 10494272.9 | 20720287.35 |
| **13S-hydroxyoctadecadienoic acid** | 10754274.22 | 7655600.355 | 7531972.751 | 7570344.452 | 7251314.966 | 10110027.3 | 10498739.28 | 7077922.54 | 6472348.65 |
| **(6Z)-Octadecenoic acid** | 39404509.97 | 41657975.83 | 9753558.649 | 8318084.493 | 37990447.68 | 9086409.92 | 45838408.81 | 9293726.92 | 35459606.88 |
| **2-Oxo-4-methylthiobutanoic acid** | 3732036.297 | 12327530.21 | 4125851.431 | 13477724.81 | 16183667.21 | 18028517.3 | 11202063.7 | 14962291.5 | 37082442.72 |
| **Hexadecanedioic acid** | 33460434.51 | 19681141.82 | 17246483.01 | 25389617.84 | 29336916.67 | 29709315.9 | 24436568.15 | 18521852.5 | 29808956.75 |
| **(2'E,4'Z,8E)-Colneleic acid** | 1021740.07 | 3812387.346 | 4413369.525 | 2811804.548 | 3545656.141 | 3934930.14 | 880173.0085 | 892305.203 | 5711099.978 |
| **Azelaic acid** | 7344678.059 | 7426667.681 | 8718285.416 | 10578852.72 | 8853516.533 | 13863018.6 | 7056062.257 | 11394383 | 7359103.599 |
| **Bovinic acid** | 8200948.027 | 16356593.15 | 4183151.487 | 12383528.43 | 12866082.58 | 4399710.5 | 6530837.439 | 4263455.67 | 19928512.52 |

**Supplementary Table 5. Predicted transcription factors of Rag GTPases.**

|  | **Prediction in Jaspar (Score)** | | | | **RNA-Seq** | | **Prediction in TFDB (Score)** | | | |  |
| --- | --- | --- | --- | --- | --- | --- | --- | --- | --- | --- | --- |
| **Name** | **RRAGA** | **RRAGB** | **RRAGC** | **RRAGD** | | **Fold Change** | **RRAGA** | **RRAGB** | **RRAGC** | **RRAGD** | |
| **SP1** | 13.369 | 14.830 | 14.830 | 16.178 | | 4.981 | 17.000 | 20.333 | 19.382 | 22.357 | |
| **SP4** | 12.657 | 14.734 | 14.734 | 15.903 | | 3.636 | 16.079 | 18.057 | 19.384 | 23.950 | |
| **SP2** | 12.478 | 14.224 | 14.966 | 15.941 | | 3.529 | 19.896 | 22.481 | 26.104 | 24.632 | |
| **ZNF384** | 15.290 | 16.357 | 16.308 | 14.345 | | 2.899 | 20.513 | 26.278 | 33.667 | 20.217 | |
| **USF1** | 12.850 | 15.361 | 12.830 | 16.210 | | 2.298 | 17.408 | 20.171 | 18.697 | 20.645 | |
| **PRDM1** | 13.785 | 14.391 | 13.165 | 15.320 | | 1.486 | 12.118 | 14.461 | 13.526 | 13.526 | |
| **USF2** | 12.797 | 17.753 | 12.305 | 15.676 | | 1.352 | 16.729 | 18.566 | 16.961 | 18.882 | |
| **E2F6** | 12.891 | 13.794 | 15.875 | 13.048 | | 1.016 | 13.674 | 14.741 | 16.379 | 14.622 | |
| **EGR1** | 11.476 | 12.147 | 15.426 | 19.087 | | 3.038 | 15.424 | 13.603 | 18.470 | 21.314 | |
| **TFAP2B** | 11.410 | 12.947 | 12.498 | 16.479 | | N/A | 13.326 | 14.798 | 13.056 | 14.449 | |
| **TFAP2C** | 10.940 | 12.758 | 11.293 | 15.721 | | 1.487 | 20.566 | 15.684 | 14.211 | 15.184 | |
| **MITF** | 11.814 | 16.107 | 11.257 | 15.612 | | 2.708 | 12.043 | 18.900 | 14.540 | 14.712 | |
| **TFAP2A** | 11.713 | 12.526 | 12.901 | 15.415 | | 1.679 | 15.237 | 15.868 | 13.605 | 16.671 | |
| **JUN** | 13.151 | 10.480 | 11.449 | 15.015 | | 0.319 | 11.980 | 12.127 | 14.195 | 15.843 | |
| **KLF4** | 10.795 | 15.008 | 11.219 | 14.861 | | 0.501 | 14.300 | 17.636 | 16.183 | 20.338 | |
| **CTCFL** | 12.964 | 10.360 | 12.549 | 14.673 | | N/A | 15.515 | 15.046 | 14.742 | 13.530 | |
| **KLF1** | 11.333 | 13.785 | 11.333 | 14.660 | | N/A | 17.882 | 14.817 | 14.000 | 18.921 | |
| **EHF** | 10.985 | 18.520 | 10.961 | 13.801 | | 3.805 | 12.795 | 18.836 | 13.192 | 16.493 | |
| **KLF5** | 12.138 | 14.917 | 10.694 | 13.577 | | 2.493 | 19.227 | 17.515 | 17.237 | 20.197 | |
| **NFYA** | 13.118 | 10.993 | 13.926 | 13.572 | | 5.066 | 14.986 | 17.013 | 19.529 | 17.106 | |
| **PAX5** | 14.774 | 11.286 | 12.613 | 13.253 | | N/A | 15.661 | 12.726 | 14.461 | 15.046 | |
| **RFX2** | 16.663 | 14.098 | 9.815 | 13.095 | | 2.221 | 17.086 | 16.842 | 16.276 | 14.618 | |
| **MAX** | 13.164 | 12.464 | 11.130 | 13.084 | | 1.331 | 18.026 | 17.776 | 17.579 | 16.382 | |
| **MXI1** | 12.142 | 11.755 | 11.515 | 12.622 | | 2.752 | 17.776 | 18.526 | 15.013 | 18.079 | |
| **RBPJ** | 11.279 | 12.760 | 12.307 | 12.582 | | 2.566 | 18.395 | 13.829 | 11.671 | 12.421 | |
| **CDX2** | 10.390 | 13.119 | 11.769 | 12.554 | | N/A | 19.697 | 12.461 | 13.290 | 13.290 | |
| **SPIB** | 11.121 | 18.968 | 10.470 | 12.521 | | 23.308 | 14.092 | 19.652 | 12.605 | 12.727 | |
| **RFX3** | 13.633 | 16.077 | 10.557 | 12.430 | | 7.506 | 17.500 | 17.212 | 12.329 | 15.368 | |
| **TFE3** | 10.867 | 18.067 | 14.178 | 12.344 | | 2.699 | 11.429 | 13.857 | 11.602 | 13.898 | |
| **PITX2** | 12.317 | 13.742 | 12.317 | 12.317 | | 1.129 | 13.418 | 13.561 | 13.418 | 13.418 | |
| **OTX2** | 11.214 | 12.672 | 11.628 | 12.205 | | N/A | 15.303 | 16.275 | 14.316 | 14.553 | |
| **MEF2A** | 14.824 | 11.808 | 13.088 | 12.008 | | 5.214 | 14.451 | 13.855 | 14.013 | 14.500 | |
| **CTCF** | 20.481 | 12.622 | 19.275 | 11.697 | | 4.767 | 22.816 | 18.987 | 17.258 | 17.303 | |
| **ETV4** | 14.706 | 14.571 | 10.252 | 11.612 | | 3.521 | 14.171 | 12.579 | 12.974 | 23.723 | |
| **GATA3** | 14.681 | 13.321 | 14.681 | 11.477 | | 2.904 | 14.327 | 15.971 | 16.316 | 14.513 | |
| **KLF6** | 11.418 | 11.502 | 11.418 | 11.342 | | 4.949 | 12.615 | 12.257 | 13.973 | 12.615 | |
| **TEAD4** | 11.895 | 10.798 | 10.765 | 11.128 | | 1.457 | 13.500 | 13.250 | 14.325 | 15.171 | |
| **GATA2** | 14.521 | 13.564 | 13.622 | 11.039 | | 2.001 | 14.491 | 13.225 | 13.833 | 13.947 | |
| **ELF1** | 12.570 | 18.376 | 12.033 | 10.714 | | 2.828 | 17.314 | 18.803 | 23.987 | 16.579 | |
| **GATA6** | 15.150 | 13.416 | 14.931 | 10.500 | | 2.659 | 15.015 | 14.763 | 16.078 | 13.402 | |
| **ASCL1** | 15.580 | 12.537 | 10.192 | 10.061 | | N/A | 15.106 | 14.409 | 14.930 | 14.535 | |
| **MEF2C** | 12.328 | 11.544 | 14.020 | 10.031 | | 6.116 | 14.105 | 15.684 | 13.092 | 11.134 | |
| **GATA4** | 13.006 | 10.770 | 12.394 | 9.978 | | N/A | 14.470 | 13.658 | 14.079 | 16.540 | |
| **IKZF1** | 10.749 | 16.524 | 10.824 | 9.822 | | 1.000 | 14.333 | 16.212 | 14.712 | 14.763 | |
| **FOXP1** | 14.274 | 15.635 | 13.100 | 9.820 | | 3.591 | 16.816 | 15.553 | 18.145 | 15.184 | |

| **Supplementary Table 6. Clinical characteristics of human specimens.** | | | | |  | | |  | | |  | |  |
| --- | --- | --- | --- | --- | --- | --- | --- | --- | --- | --- | --- | --- | --- |
| **Pathological number** | **Types** | | **Grade** | **Subtypes** | | **Age** | | | |  |  |  |  |
| 201723889 | Invasive ductal carcinoma | | WHO I | Luminal B | | 80 | | | |  |  |  |  |
| 201702945 | Invasive ductal carcinoma | | WHO III | TNBC | | 64 | | | |  |  |  |  |
| 201618495 | Invasive ductal carcinoma | | WHO II | Luminal A | | 39 | | | |  |  |  |  |
| 201615795 | Invasive ductal carcinoma | | WHO III | HER-2 | | 37 | | | |  |  |  |  |
| 201616852 | Invasive ductal carcinoma | | WHO II | Luminal B | | 50 | | | |  |  |  |  |
| 201618494 | Invasive ductal carcinoma | | N/A | Luminal B | | 53 | | | |  |  |  |  |
| 201636232 | Ductal carcinoma in situ | | WHO I | Luminal A | | 48 | | | |  |  |  |  |
| 201710861 | Invasive ductal carcinoma | | WHO II | Luminal B | | 48 | | | |  |  |  |  |
| 201712301 | Invasive ductal carcinoma | | WHO I | Luminal B | | 58 | | | |  |  |  |  |
| 201718292 | Invasive ductal carcinoma | | WHO III | Luminal B | | 45 | | | |  |  |  |  |
| 201618645 | Ductal carcinoma in situ | | N/A | HER-2 | | 57 | | | |  |  |  |  |
| 201726333 | Invasive ductal carcinoma | | WHO III | Luminal B | | 62 | | | |  |  |  |  |
| 201729845 | Invasive ductal carcinoma | | WHO I | Luminal B | | 46 | | | |  |  |  |  |
| 201731001 | Invasive ductal carcinoma | | WHO III | Luminal B | | 37 | | | |  |  |  |  |
| 201608752 | Invasive ductal carcinoma | | WHO II | Luminal A | | 69 | | | |  |  |  |  |
|  |  |  | | | | |  | |  | | |  | |
|  |  |  | | | | |  | |  | | |  | |
|  | **Breast cancer cases** |  | | | | |  | |  | | |  | |
|  | n (%) |  | | | | |  | |  | | |  | |
| **Sample number (n)** | 15 |  | | | | |  | |  | | |  | |
| **Age (mean ± SD)** | 52.87 ± 12.27 |  | | | | |  | |  | | |  | |
| **Tumor grade/mBR grade I** | 4（26.7） |  | | | | |  | |  | | |  | |
| **Tumor grade/mBR grade II** | 4 （26.7） |  | | | | |  | |  | | |  | |
| **Tumor grade/mBR grade III** | 5（33.3） |  | | | | |  | |  | | |  | |
| **ER positivity** | 3（20） |  | | | | |  | |  | | |  | |
| **PR positivity** | 3（20） |  | | | | |  | |  | | |  | |
| **HER2 positivity** | 11（73.3） |  | | | | |  | |  | | |  | |
|  |  |  | | | | |  | |  | | |  | |
